# Effects of the Chinese herbal formula San-Huang Gu-Ben Zhi-Ke treatment on stable chronic obstructive pulmonary disease: a randomized, double-blind, placebo-controlled trial

**DOI:** 10.3389/fphar.2023.1164818

**Published:** 2023-06-27

**Authors:** Tianyi Lyu, Demin Li, Xiang Lei, Yuteng Zhang, Shilei Cheng, Xinyang Shu, Hongchun Zhang

**Affiliations:** ^1^ School of Acupuncture-Moxibustion and Tuina, Beijing University of Chinese Medicine, Beijing, China; ^2^ Department of Traditional Chinese Medicine for Pulmonary Diseases, National Center for Respiratory Medicine, National Clinical Research Center for Respiratory Diseases, Institute of Respiratory Medicine, Center of Respiratory Medicine, China-Japan Friendship Hospital, Chinese Academy of Medical Sciences, Beijing, China; ^3^ Beijing Qi-Huang Technology Co., Ltd., Beijing, China; ^4^ Beijing University of Chinese Medicine, Beijing, China

**Keywords:** chronic obstructive pulmonary disease, traditional Chinese medicine, San-Huang Gu-Ben Zhi-Ke, clinical trials, subgroup analysis

## Abstract

**Objective:** The aim of this study was to evaluate the efficacy and safety of the Chinese herbal formula San-Huang Gu-Ben Zhi-Ke (SHGBZK) as a treatment for patients with stable chronic obstructive pulmonary disease (COPD) diagnosed with lung-spleen Qi deficiency.

**Method:** A randomized, double-blind, placebo-controlled trial was designed. 98 adults aged between 40 and 80 years with stable COPD diagnosed with lung-spleen Qi deficiency were included. All participants received basic treatment for COPD. Patients in the experimental group took SHGBZK, while the control group took placebo. The primary outcome was the frequency of acute exacerbation. The secondary outcomes were lung function, symptom score, exercise capacity and quality of life.

**Results:** Of 98 patients who underwent randomization, 50 patients in the SHGBZK group and 48 in the placebo group were included in the full analysis set. After 24-week therapy and 28-week follow-up, patients in treatment group had significant improvements in symptom, exercise capacity and quality of life. After Subgroup analysis, the frequency of acute exacerbation in patients with a COPD Assessment Test (CAT) score of at least 10 or a modified Medical Research Council (mMRC) score of at least 2 was significantly lower in the SHGBZK group than in the placebo group. Lung function in patients with frequent exacerbation was significantly higher in the SHGBZK group than in the placebo group. The incidence of adverse events was generally similar in the two groups.

**Conclusion:** SHGBZK had beneficial effects on symptom, exercise capacity and quality of life in stable COPD patients. SHGBZK also had the potential to reduce the frequency of exacerbation and improve lung function in specific groups of COPD patients.

**Clinical Trial Registration:** https://www.chictr.org.cn/showproj.html?proj=26933, identifier ChiCTR1800016349

## Introduction

Chronic obstructive pulmonary disease (COPD) is a highly prevalent disease, with a prevalence of 174.5 million adults worldwide and 99.9 million adults in China. The morbidity of COPD will rise steadily in the next 40 years 5.4 million death is estimated to be due to COPD globally in 2060 (GBD, 2015 Chronic Respiratory Disease Collaborators, 2017; Wang et al., 2018). Considering its high rates of morbidity and disability, COPD poses a serious threat to human health.

In addition to pulmonary symptoms, the most common complications of COPD include cardiovascular disease, diabetes, asthma, and anemia, and the majority of patients have one or two comorbidities, adding to the burden on society and the medical system (Mannino et al., 2015). Moreover, most COPD patients suffer from mental diseases that have a negative impact on their quality of life (Christiansen et al., 2023). Given that COPD is a preventive disease, effective treatment can delay the recurrence and progression of the disease.

Pharmacological interventions, mainly bronchodilators and glucocorticoid, have been proven to be effective in relieving symptoms, improving quality of life and exercise capacity, and reducing risk of exacerbation in patients with stable COPD (Ritchie et al., 2020). However, even after drug therapies, some patients with stable COPD experience frequent acute exacerbations and have decreased lung function (Contoli et al., 2020). Existing treatments cannot fundamentally improve patients’ prognosis, delay disease progression, or reduce mortality rates. Therefore, alternative and effective therapies are still required to improve management of stable COPD.

“Qi” is a unique concept in Traditional Chinese medicine (TCM). In TCM, lung can govern Qi and control breathing while spleen has the ability to digest, acquire, and transmit energy (Qi) from food, hence boosting lung Qi (Liu et al., 2020). If the lung becomes weak, the spleen suffers and cannot function well. Therefore, lung-spleen Qi deficiency is one of the most common TCM symptom patterns identified in patients with stable COPD (Li et al., 2019).

SHGBZK is a Chinese herbal formula developed by Professor Chao Enxiang, a great doctor of Chinese medicine with more than 50 years of clinical experience. Preliminary clinical data demonstrated that it has good clinical efficacy in COPD patients diagnosed with lung-spleen Qi deficiency (Yin et al., 2001). In addition, SHGBZK is composed of seven Chinese traditional medicinals including *Astragalus propinquus* (Huang Qi), *Polygonatum sibiricum* (Huang Jing), *Pericarpium citri reticulatae* (Chen Pi), *Stemona japonica* (Bai Bu), *Schisandra chinensis* (Wu Wei Zi) *Paeonia lactiflora* Pall (Chi Shao) and *Scutellaria baicalensis* Georgi (Huang Qin). Animal experiments have revealed that SHGBZK has a mucosal immune barrier function and can maintain airway wall integrity, reduce inflammatory cell infiltration, promote inflammatory damage repair, and alleviate airway narrowing (Tang et al., 2018). Network pharmacology demonstrated that *A. propinquus* (Huang Qi) and *Stemona japonica* (Bai Bu) had the potential to improve inflammatory response, oxidative stress, and angiogenesis in the treatment of COPD (Hu et al., 2020). *Scutellaria baicalensis Georgi* (Huang Qin), an important component of SHGBZK, has also been shown to exert anti-inflammatory and antioxidant effects in COPD rats (Wang et al., 2020). However, there is limited evidence for randomized controlled trials on SHGBZK, thus, it is difficult to fully reflect the efficacy, characteristics and advantages of SHGBZK. Therefore, we performed a prospective randomized, double-blind, placebo-controlled trial to evaluate the efficacy and safety of SHGBZK for further research into developing a new, safe, and effective method for the treatment of stable COPD.

## Methods

Our full trial protocol has been reported elsewhere (Zu et al., 2019). The methods reported here follow the Consolidated Standards of Reporting Trials (Schulz et al., 2010).

## Design

We conducted a randomized, double-blind, placebo-controlled trial. Ninety-eight patients with stable COPD were randomly assigned to two treatment groups (SHGBZK treatment, *N* = 50; placebo treatment, *N* = 48). The two groups received basic treatment for COPD according to the 2017 GOLD Guidelines for Chronic Obstructive Pulmonary Disease. Both groups received a 24-week intervention and patient status was assessed at 24 weeks and then 28 weeks after treatment. The study trial was registered on the Chinese Clinical Trial Registry (ChiCTR1800016349).

## Participants and recruitment

Participants in this study were adults aged between 40 and 80 years with stable COPD. Inclusion criteria included: 1) the diagnostic criteria in the “Guidelines for the diagnosis and treatment of chronic obstructive pulmonary disease” (2013 revision) (Chronic obstructive pulmonary disease group et al., 2013); 2) patients with stable symptoms such as cough, sputum production, or breathlessness in the past 4 weeks; 3) patients with high risk assessment of AECOPD (two or more AECOPD occurrences or hospitalized at least once due to AECOPD in the past year); 4) patients diagnosed with TCM syndrome pattern of lung -spleen Qi deficiency by Guidelines for TCM Diagnosis and Treatment of Chronic Obstructive Pulmonary Disease (2011 edition) (Professional Committee of Pulmonary Diseases, 2012); 5) patients with informed signed consent and voluntary participation in the study.

Exclusion criteria included: 1) patients diagnosed with pneumonia and/or moderate to severe AECOPD in the past 4 weeks; 2) patients accepted pneumonectomy in the past or lung volume reduction surgery in the 12 months before screening; 3) patients accepted long-term oxygen therapy (time>15 h/day) or mechanical aerator; 4) patients with a history of asthma, active tuberculosis, lung cancer, bronchiectasis, pulmonary embolism, pulmonary heart disease, interstitial lung disease, or other active diseases; 5) patients with lower extremity activity limitation and unable to complete the 6-min walk test; 6) patients diagnosed with serious hypertension, diabetes, tumors, or primary heart, liver, kidney, or blood system disease; 7) scr exceeds the upper limit of the reference value by 1.5 times, or AST/ALT ratio ≥2 times the upper limit of the reference value; 8) patients with congenital or acquired immunodeficiency disease; 9) patients who are known or suspected of a history of alcohol or drug abuse; 10) patients with confusion, dementia, or any kind of mental illness; pregnant or breast-feeding women; 11) patients who are allergic to the SHGBZK or who take glucocorticoids on a regular basis; 12) patients enrolled in other clinical trials during the previous 3 months; 14) anyone researchers believe should not participate in the clinical trial.

All patients signed informed consent before inclusion. The study has been approved by the Ethics Research Committees of the China-Japanese Friendship Hospital with identifier 2018-57-K41-1. Any revisions of the study protocol were submitted to the ethics committee. The research participants were recruited from either out-patient department or open recruitment at China-Japanese Friendship Hospital between November 2018 and June 2021.

## Intervention

The two groups received basic treatment for COPD according to the 2017 GOLD Guidelines for Chronic Obstructive Pulmonary Disease (Vogelmeier et al., 2017). Patients adhered to their previous treatment regimen and were given the treatment when AECOPD occurred during the study. Patients in the experimental group took SHGBZK, while those in the control group took SHGBZK placebo. The TCM granules were compound preparations of Chinese herbs; its main components are shown in Supplementary Table S1. Each bag of SHGBZK granules (batch number 180606) contained 3 g. The components of the TCM granules were produced and packed by An Hui Ji Ren Pharmaceutical Co., Ltd. according to Good Manufacturing Practice (approval number AH20160363), Anhui, China. The test results of drug quality were consistent with the required quality standards. Each type of granule was given orally, four bags each time, three times a day for 24 weeks. Patients needed to take the medication as directed by the doctor. The use of glucocorticoids, antibiotics, mucolytic agents, and antitussive agents was prohibited during the study unless AECOPD occurred. Oral or external Chinese medicine preparations with the effect of tonifying the spleen and lung were prohibited during the trial period.

Patients were given a daily diary to record their trial medication compliance, as well as use of any other therapies and occurrence of adverse events. Patients were asked to return their medication bags monthly during the treatment period so that left-over capsules could be counted and participant adherence could be monitored.

## Outcomes and procedures

### Primary outcome

The frequency of acute exacerbation was the primary outcome. AECOPD was characterized by increased respiratory symptoms and medication changes. Its reduction was a major goal of COPD management and an important indicator for evaluating the treatments. AECOPD was defined as the presence of at least two major symptoms, or one major symptom plus more than one minor symptoms. Major symptoms were increased dyspnea, increased sputum volume, purulent sputum. Minor symptoms were upper respiratory tract infection, unexplained fever, and wheezing. If the time between two acute exacerbations was less than 1 week and the acute exacerbation lasted at least 2 days, it was considered one acute exacerbation event. The frequency of AECOPD occurrences during the 24-week treatment period and 28-week follow-up was counted.

## Secondary outcomes

### Lung function

Forced vital capacity (FVC), forced expiratory volume in 1 s (FEV1), forced expiratory volume in 1 s (FEV1% pred), FEV1/FVC, maximum expiratory mid-flow (MMEF), and peak expiratory flow (FEF) were tested. A positive change from baseline in these indicators indicated an improvement in lung function.

### Symptom and quality of life

The COPD Assessment Test (CAT) was adopted. The CAT is a self-complete questionnaire with eight items, each formatted as a 6-point semantic differential scale ranging from 0 to 5. CAT scores range from 0 to 40. Higher scores denote a more severe impact on a patient’s quality of life.

The Modified Medical Research Council (mMRC) scale by the American Thoracic Society were assessed to evaluate the level of dyspnea. The mMRC scale is a simple grading system that scores from 0 (normal) to 4 (severe).

The TCM symptom score scale for patients with stable COPD diagnosed with lung-spleen Qi deficiency was adopted. The TCM symptom score scale is scored from 0 (normal) to 22 (severe). The TCM symptom score is shown in Supplementary Table S2.

Patients were invited to complete the questionnaires through face-to-face survey. Patients answered each question and checked the most appropriate opinion (a specific score) with regards to their standards, hopes, pleasures, and concerns. Meanwhile, an investigator was assigned to each center’s office to assist patients and to review each completed questionnaire to ensure that patients answered all of the questions.

### Exercise capacity

The 6-min walking distance (6MWD) was used to evaluate the distance a person can walk on a flat surface in 6 min to assess their exercise capacity. The BODE index was also used to assess exercise capacity. BODE stands for Body mass index, airflow Obstruction, Dyspnea and Exercise capacity. BODE scores ranged from 0 to 40 and are further quartilized as follows: quartile 1 (a score of 0–2 points), quartile 2 (a score of 3–4 points), quartile 3 (a score of 5–6 points), and quartile 4 (a score of 7–10 points). The higher the level rise, the worse the patient’s condition becomes.

### Concomitant medication status

Drug therapies used to treat COPD during the study were recorded.

### Mortality

All-cause mortality and COPD mortality were calculated for the subjects during the study.

### Safety

Routine blood, urine and stool tests, liver and kidney function tests, and an electrocardiogram were performed. Adverse events were recorded at any time during the treatment period and follow-up period. Adverse events were recorded and graded in detail throughout the study. If a severe adverse event occurred, participants were given all necessary treatment, and the event had to be reported within 24 h to the trial’s leader, ethics committees and sponsors.

### Screening and run-in, baseline, treatment periods, and endpoint

Adverse events, physical examination, AECOPD situation, mMRC, CAT, and TCM symptom score scale were recorded at baseline (week 0) and every 4 weeks during the study period. The 6MWD and BODE were recorded at week 0, 4, 12, 24, 32, and 52. Lung function were observed at week 0, 24, and 52. Safety were measured at week 0, 12, and 24, not including adverse events and the physical examination.

### Sample size

A total of 100 patients were enrolled in this study with 50 in each group. The frequency of AECOPD was the primary outcome. According to previous studies, the exacerbation frequency increased 1.17 times each year when receiving conventional medicine, 0.97 times each year when receiving TCM, and 0.68 times each year when receiving both conventional medicine and TCM. Assume that promotional value is achieved only when the exacerbation frequency decreases at least once for each patient every 6 months. The standard deviation (SD) value is 1.25 times per year, the two-sided α is 0. 05, and β is 0. 10. Based on the formula:

$$\left(\frac{2{\left(\mu \alpha +\mu \beta \right)}^{2}\sigma^{2}}{\delta^{2}}\right)$$

of the comparison between the means of the two samples, the sample size in each group is 40. Considering a 20% dropout rate over the course of the study, 50 patients were enrolled in each group and the total sample size were 100.

## Randomization and masking

### Randomization

The block randomization method was used. The length of the block was 4 and SAS9.4 (SAS Institute Inc., Cary, NC, United States) was used to generate a randomization sequence for 104 subjects (test group, control group) according to a 1:1 ratio and list the treatment allocation corresponding to serial numbers 001–104 (that is, a random coding table). But only 50 patients were recruited in each group. To ensure that the placebo looked, smelled, and tasted like SHGBZK, it was made of SHGBZK (5%) and dextrin (95%). Both researchers and participants did not know the assignment. The randomization sequence table was kept in a file. The method, process, group setting, and grouping result of the randomization sequence were recorded so it could be checked when necessary. Information on intervention assignments were kept in the third consulting center of biomedical statistics.

### Blinding

Design: In this study, two stage blinding was used. The first stage blinding was represented by group A and B. The second stage blinding was represented by the corresponding test drug and placebo.

Blinding management and preservation: Blinding was carried out by the statistical unit. The clinical trial unit and the statistical analysis unit were deposited in accordance with the relevant regulations after the blinding was sealed. The process of drug coding were written by the blinder and saved.

Unblinding under emergency: If an adverse event occurred during the study, the main investigator decided whether or not to unblind according to the subject. The investigator needed to record the time, location, and cause of the unblinding in the medical record and Case Report Form (CRF) (the group information after unblinding should not be recorded in the CRF).

### Statistical analysis

All data was analyzed by an independent statistician using SAS 9.4. All analyses were by intention to treat (ITT). For all analyses, *p* < 0.05 was considered statistically significant. Measurement data were presented as number of cases, mean, standard deviation, median, quartile or percentage. *t*-test, signed rank sum test or chi-square test were used to compare the difference between the two groups, or pre-treatment and post-treatment within one group. Analysis of covariance was used to compare the differences of center effector and other confounding factors. Subgroup analysis was performed in frequency of AECOPD and lung function according to sex (male vs. female), CAT score (<10 vs.≥10), mMRC scale (<2 vs. ≥2), GOLD grade (1–2 vs. 3–4) and history of frequent acute exacerbation (≤2 vs. > 2).

### Withdrawal, dropout, and discontinuation

Participants were free to withdraw at any time during the trial. Participants who wish to withdraw were offered the option to cease trial medication but continued to attend scheduled visits for outcome measurements. Participants who withdraw were traced to investigate the reason for withdrawal. Participants were advised to discontinue the treatment if there was a product-related serious adverse event or if the participant was not compliant with the study requirements. Intention-to-treat analysis were performed on missing data from discontinuers with the last observation carried forward method.

## Results

### Baseline characteristic

**FIGURE 1:**
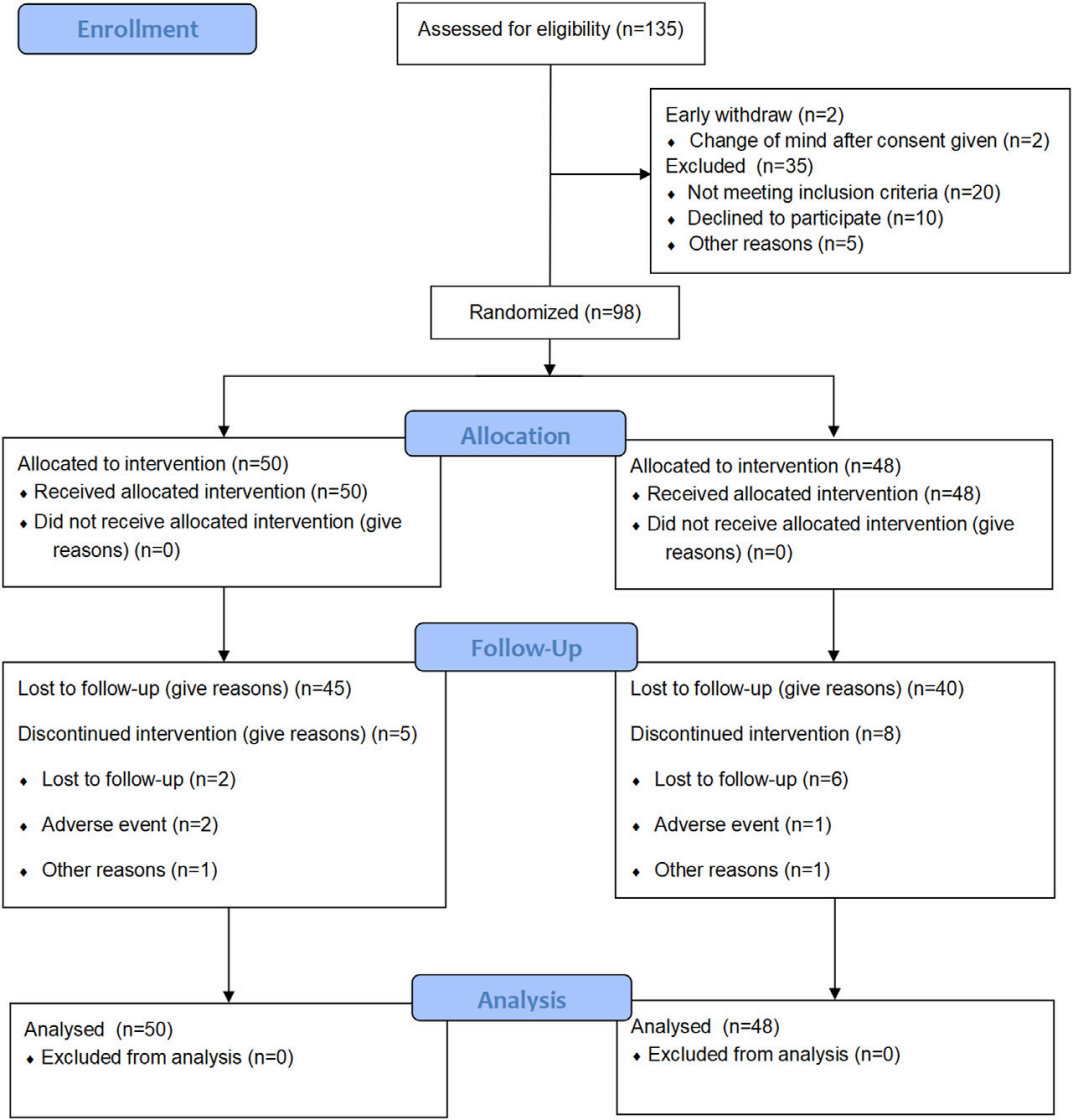
Flowchart of this study.

There were 135 patients diagnosed as stable COPD for screening from November 2018 to June 2021. A total of 100 patients meeting the inclusion criteria were enrolled finally. Two patients withdraw after consent given. Other eligible patients were randomly assigned to the SHGBZK group and the placebo treatment group. During follow-up, five subjects in the SHGBZK group and eight subjects in the placebo group dropped out. Finally, 50 subjects in the SHGBZK group and 48 subjects in the placebo group were analyzed (Figure 1). There were no significant differences in demographic characteristics and vital signs between the two groups (Table 1).

**TABLE 1:** Characteristics of the patients at baseline.

Characteristic	SHGBZK group (*N* = 50)	Placebo group (*N* = 48)	*p*-value
Age (years)	66.34 (6.61)	66.81 (6.63)	0.7248
Male sex (%)	42 (84.00)	37 (77.08)	0.3866
Smoking status			0.2986
Never smoked	11 (22.00)	13 (27.08)	
Current smoking	12 (24.00)	17 (35.41)	
Former smoking	27 (54.00)	18 (37.50)	
Duration of COPD (months)	67.90 (57.73)	90.44 (110.16)	0.2087
GOLD stage (%)			
1	8 (16.00)	13 (27.08)	0.5038
2	22 (44.00)	19 (39.58)	
3	17 (34.00)	15 (31.25)	
4	3 (6.00)	1 (2.08)	
CAT score	10.38 (5.80)	10.54 (6.24)	0.8946
mMRC scale score			0.3717
0 (%)	7 (14.00)	8 (16.67)	
1 (%)	15 (30.00)	14 (29.17)	
2 (%)	15 (30.00)	20 (41.67)	
3 (%)	12 (24.00)	6 (12.50)	
4 (%)	1 (2.00)	0 (0.00)	
Lung function			
FEV1	1.47 (0.65)	1.69 (0.72)	0.1325
FVC	2.86 (0.82)	3.19 (0.92)	0.0794
FEV1%	50.64 (14.36)	51.95 (12.86)	0.6477
PEF	4.11 (1.81)	4.78 (2.04)	0.0983
MMEF	0.69 (0.55)	0.71 (0.48)	0.8549

Note. Data are shown as mean ± SD, or n (%). *p* values were calculated by *t*-test, signed rank sum test or chi-square test. *p < 0.05.

## Primary outcome

The frequency of acute exacerbation decreased in both groups in the intervention stage (the first 24 weeks) and the follow-up stage (week 25–52). This decrease could also be observed over the whole trial (52 weeks). The SHGBZK group had a lower mean frequency of acute exacerbation than the placebo group. However, no significant between-group differences were observed (*p* = 0.1586, *p* = 0.0664, *p* = 0.1034). The results were shown in Table 2.

**TABLE 2:** Comparison of the frequency of acute exacerbations.

Follow-up	SHGBZK		Placebo		*p*-value
	N	Mean (SD)	N	Mean (SD)	
Baseline	49 (1)	2.00 (1.73)	47 (1)	2.04 (1.76)	0.9051
Week 24	50 (0)	0.02 (0.06)	48 (0)	0.05 (0.13)	0.1586
Week 52	50 (0)	0.10 (0.36)	48 (0)	0.38 (1.12)	0.1034
Week 24–52	50 (0)	0.01 (0.04)	48 (0)	0.03 (0.09)	0.0664

Note. Data are shown as mean ± SD. *p* values were calculated by *t*-test, signed rank sum test or chi-square test. *p < 0.05.

## Secondary outcomes

### Lung function

SHGBZK ameliorated the annual decline in the FVC, comparing with placebo treatment. SHGBZK also improved FEV1, FEV1%, PEF during week 52. However, there were no significant between-group differences in any parameters of lung function at any time (*p* > 0.05). The results were shown in Table 3.

**TABLE 3:** Difference of lung function between the two groups at different time points.

Variable	SHGBZK		Placebo		*p*-value
	N	Mean (SD)	N	Mean (SD)	
FEV1					
T0	44 (6)	1.47 (0.65)	47 (1)	1.69 (0.72)	0.1325
T1	48 (2)	1.48 (0.69)	48 (0)	1.70 (0.67)	0.1271
T2	48 (2)	1.51 (0.77)	48 (0)	1.62 (0.66)	0.4565
T1-T0	44 (6)	−0.03 (0.26)	47 (1)	−0.01 (0.24)	0.7664
T2-T0	44 (6)	0.01 (0.47)	47 (1)	−0.09 (0.18)	0.1829
FVC					
T0	44 (6)	2.86 (0.82)	47 (1)	3.19 (0.92)	0.0794
T1	48 (2)	2.91 (0.87)	48 (0)	3.16 (0.89)	0.1709
T2	48 (2)	2.83 (0.92)	48 (0)	3.08 (0.91)	0.1769
T1-T0	44 (6)	−0.04 (0.36)	47 (1)	−0.04 (0.31)	0.9400
T2-T0	44 (6)	−0.09 (0.40)	47 (1)	−0.12 (0.35)	0.6747
FEV1%					
T0	44 (6)	50.64 (14.36)	47 (1)	51.95 (12.86)	0.6477
T1	48 (2)	49.79 (13.52)	48 (0)	53.24 (12.69)	0.2009
T2	48 (2)	51.35 (13.26)	48 (0)	51.82 (12.68)	0.8621
T1-T0	44 (6)	−0.42 (7.43)	47 (1)	1.01 (8.76)	0.4047
T2-T0	44 (6)	1.00 (10.75)	47 (1)	−0.45 (4.32)	0.3960
PEF					
T0	44 (6)	4.11 (1.81)	47 (1)	4.78 (2.04)	0.0983
T1	48 (2)	4.40 (1.99)	48 (0)	4.90 (2.00)	0.2266
T2	35 (15)	4.40 (2.12)	34 (14)	4.69 (2.20)	0.5791
T1-T0	44 (6)	0.15 (0.85)	0.11 (0.88)	0.12 (0.89)	0.8237
T2-T0	35 (15)	0.19 (1.03)	34 (14)	0.00 (0.61)	0.3668
MMEF					
T0	43 (7)	0.66 (0.53)	47 (1)	0.71 (0.48)	0.6741
T1	48 (2)	0.60 (0.43)	47 (1)	0.73 (0.46)	0.1623
T2	35 (15)	0.57 (0.35)	34 (14)	0.65 (0.50)	0.4324
T1-T0	43 (7)	−0.11 (0.45)	46 (2)	0.01 (0.29)	0.1699
T2-T0	31 (19)	−0.13 (0.51)	34 (14)	−0.04 (0.15)	0.2955

Note. Data are shown as mean ± SD. *p* values were calculated by *t*-test, signed rank sum test or chi-square test. *p < 0.05. T0: baseline, T1: week 24, T2: week 52.

### Quality of life

**FIGURE 2:**
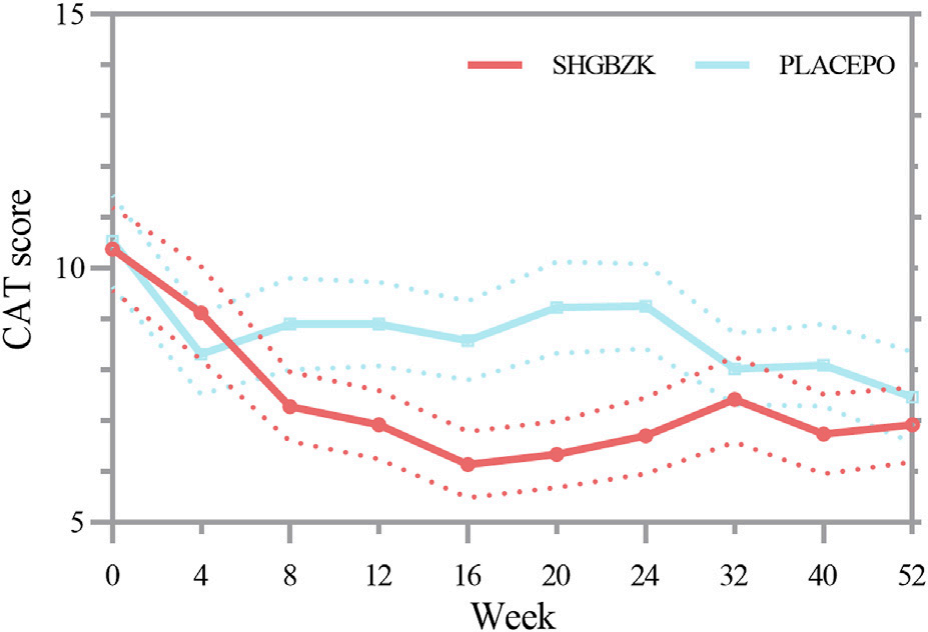
Measures of CAT score CAT score in the SHGBZK group and place at week 0, 4, 12, 32, and 52.

**FIGURE 3:**
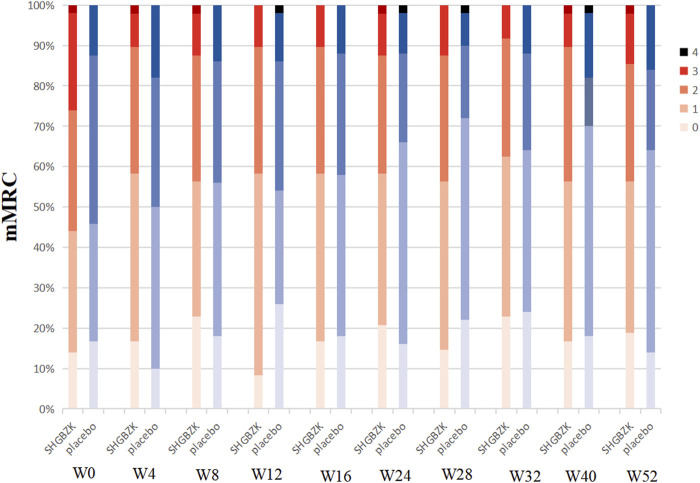
Measures of mMRC scale mMRC in SHGBZK group and placebo at week 0, 4, 8, 16, 24, 28, 32, 40, and 52.

SHGBZK was more effective than placebo with regard to mMRC scale during week 24 (*p* = 0.0181) and CAT scores during week 16, week 20, week 24 (*p* = 0.0428, *p* = 0.0272, *p* = 0.0423). There were no significant between-group differences in other time points (*p* > 0.05). Details are provided in Tables 4, 5, Figures 2, 3.

**TABLE 4:** Difference of mMRC scores between the two groups at different time points.

Follow-up	SHGBZK (*N* = 50)					Placebo group (*N* = 48)					*p*-value
	0	1	2	3	4	0	1	2	3	4	
T0	7 (14.00)	15 (30.00)	15 (30.00)	12 (24.00)	1 (2.00)	8 (16.67)	14 (29.17)	20 (41.67)	6 (12.50)	0 (0.00)	0.3717
T1	5 (10.00)	20 (40.00)	16 (32.00)	9 (18.00)	0 (0.00)	8 (16.67)	20 (41.67)	15 (31.25)	4 (8.33)	1 (2.08)	0.2522
T1-T0											0.9238
T2	9 (18.00)	19 (38.00)	15 (30.00)	7 (14.00)	0 (0.00)	11 (22.92)	16 (33.33)	15 (31.25)	5 (10.42)	1 (2.08)	0.7721
T2-T0											0.5259
T3	13 (26.00)	14 (28.00)	16 (32.00)	6 (12.00)	1 (2.00)	4 (8.33)	24 (50.00)	15 (31.25)	5 (10.42)	0 (0.00)	0.6510
T3-T0											0.0570
T4	9 (18.00)	20 (40.00)	15 (30.00)	6 (12.00)	0 (0.00)	8 (16.67)	20 (41.67)	15 (31.25)	5 (10.42)	0 (0.00)	0.9940
T4-T0											0.4821
T5	8 (16.00)	25 (50.00)	11 (22.00)	5 (10.00)	1 (2.00)	10 (20.83)	18 (37.50)	14 (29.17)	5 (10.42)	1 (2.08)	0.8243
T5-T0											0.3666
T6	11 (22.00)	25 (50.00)	9 (18.00)	4 (8.00)	1 (2.00)	7 (14.58)	20 (41.67)	15 (31.25)	6 (12.50)	0 (0.00)	0.1349
T6-T0											0.0181*
T7	12 (24.00)	20 (40.00)	12 (24.00)	6 (12.00)	0 (0.00)	11 (22.92)	19 (39.58)	14 (29.17)	4 (8.33)	0 (0.00)	0.9791
T7-T0											0.2521
T8	9 (18.00)	26 (52.00)	6 (12.00)	8 (16.00)	1 (2.00)	8 (16.67)	19 (39.58)	16 (33.33)	4 (8.33)	1 (2.08)	0.4918
T8-T0											0.1267
T9	7 (14.00)	25 (50.00)	10 (20.00)	8 (16.00)	0 (0.00)	9 (18.75)	18 (37.50)	14 (29.17)	6 (12.50)	1 (2.08)	0.8450
T9-T0											0.3533

Note. Data are shown n (%). *p* values were calculated by *t*-test, signed rank sum test or chi-square test. *p < 0.05. T0: baseline, T1: week 4, T2: week 8, T3: week 12, T4: week 16, T5: week 20, T6: week 24, T7: week 32, T8: week 40, T9: week 52.

Bold values means significant difference.

**TABLE 5:** Difference of CAT scores between the two groups at different time points.

Follow-up	SHGBZK		Placebo		*p*-value
	N	Mean (SD)	N	Mean (SD)	
T0	50 (0)	10.38 (5.80)	48 (0)	10.54 (6.24)	0.8946
T1	50 (0)	9.12 (6.51)	48 (0)	8.31 (5.51)	0.5101
T1-T0	50 (0)	−1.26 (5.45)	48 (0)	−2.23 (3.86)	0.3136
T2	50 (0)	7.28 (4.84)	48 (0)	8.90 (6.26)	0.1549
T2-T0	50 (0)	−3.10 (5.38)	48 (0)	−1.65 (5.10)	0.1733
T3	50 (0)	6.92 (4.80)	48 (0)	8.90 (5.72)	0.0665
T3-T0	50 (0)	−3.46 (5.90)	48 (0)	−1.65 (5.47)	0.1180
T4	50 (0)	6.14 (4.60)	48 (0)	8.58 (5.38)	0.0174*
T4-T0	50 (0)	−4.24 (5.85)	48 (0)	−1.96 (5.11)	0.0428*
T5	50 (0)	6.34 (4.59)	48 (0)	9.23 (6.22)	0.0101*
T5-T0	50 (0)	−4.04 (6.21)	48 (0)	−1.31 (5.81)	0.0272*
T6	50 (0)	6.70 (5.35)	48 (0)	9.25 (5.83)	0.0263*
T6-T0	50 (0)	−3.68 (6.48)	48 (0)	−1.29 (4.86)	0.0423*
T7	50 (0)	7.42 (5.98)	48 (0)	8.02 (4.84)	0.5867
T7-T0	50 (0)	−2.96 (7.47)	48 (0)	−2.52 (5.03)	0.7346
T8	50 (0)	6.74 (5.54)	48 (0)	8.10 (5.63)	0.2299
T8-T0	50 (0)	−3.64 (6.94)	48 (0)	−2.44 (4.83)	0.3237
T9	50 (0)	6.92 (5.22)	48 (0)	7.46 (6.21)	0.6430
T9-T0	50 (0)	−3.46 (6.38)	48 (0)	−3.08 (5.46)	0.7548

Note. Data are shown as mean ± SD. *p* values were calculated by *t*-test, signed rank sum test or chi-square test. *p < 0.05. T0: baseline, T1: week 4, T2: week 8, T3: week 12, T4: week 16, T5: week 20, T6: week 24, T7: week 32, T8: week 40, T9: week 52.

Bold values means significant difference.

### 6-min walking distance

**FIGURE 4:**
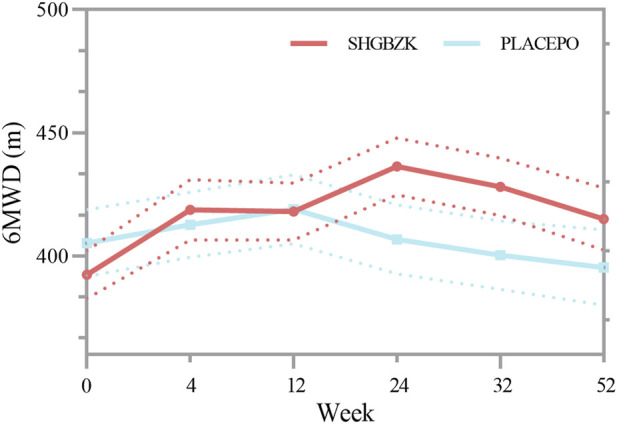
Measures of 6MWD 6MWD in the SHGBZK group and placebo at week 0, 4, 12, 24, 32, and 52.

No significant difference was observed in the mean values of 6MWD of the two groups during week 4 and week 12 (*p* = 0.128, *p* = 0.367). During week 24, week 32, and week 52, the mean value of 6MWD was significantly higher in SHGBZK group than in placebo group (*p* = 0.0029, *p* = 0.0078, *p* = 0.0370). There were no significant between-group differences in BODE index during week 24 and week 52. The results are shown in Table 6, Supplementary Table S3 and Figure 4.

**TABLE 6:** Difference of 6MWD between the two groups at different time points.

Follow-up	SHGBZK		Placebo		*p*-value
	N	Mean (SD)	N	Mean (SD)	
T0	50 (0)	392.34 (68.46)	48 (0)	405.19 (94.10)	0.4417
T1	50 (0)	418.70 (86.67)	48 (0)	412.67 (91.54)	0.7383
T1-T0	50 (0)	26.36 (61.08)	48 (0)	9.11 (48.26)	0.1276
T2	50 (0)	418.04 (81.80)	48 (0)	418.94 (97.38)	0.9606
T2-T0	50 (0)	25.70 (62.25)	48 (0)	15.51 (46.75)	0.3666
T3	50 (0)	436.40 (82.20)	48 (0)	406.67 (97.23)	0.1049
T3-T0	50 (0)	44.06 (63.60)	48 (0)	2.98 (68.56)	0.0029*
T4	50 (0)	428.04 (83.03)	48 (0)	400.27 (96.31)	0.1291
T4-T0	50 (0)	35.70 (71.39)	48 (0)	−3.55 (70.84)	0.0078*
T5	50 (0)	414.98 (88.97)	48 (0)	395.33 (105.75)	0.3214
T5-T0	50 (0)	22.64 (64.38)	48 (0)	−8.60 (80.60)	0.0370*

Note. Data are shown as mean ± SD, or n (%). *p* values were calculated by *t*-test, signed rank sum test or chi-square test. *p < 0.05. T0: baseline, T1: week 4, T2: week 12, T3: week 24, T4: week 32, T5: week 52.

Bold values means significant difference.

### TCM symptom score

The TCM symptom scores between the two groups were shown in Supplementary Table S4. TCM symptom scores of SHGBZK group continued to decrease at all time points. The SHGBZK group had a significantly lower TCM symptom score than the placebo treatment group during week 20 and week 24 (*p* = 0.0212, *p* = 0.0098).

### Concomitant medications

There were no significant differences in concomitant medications between the two groups (*p* ≥ 0.05). In the SHGBZK group, 46 patients (92.0%) were treated with other therapies. In the placebo treatment group, 40 patients (83.3%) were treated with other therapies. Details are provided in Supplementary Table S5.

### Evaluation of safety

There were no significant between-group differences in routine blood, urine and stool tests, liver and kidney function tests, and electrocardiogram in each group before and after treatment. Incidence of adverse events, serious adverse events, and death were recorded during treatment and follow-up, and there was no significant difference between the two groups (*p* > 0.05). In the SHGBZK group, 1 case had serious adverse events and 22 cases had serious adverse events. In the placebo treatment group, 21 cases had serious adverse events and 1 case dropped out due to adverse events. Details are provided in Supplementary Table S6.

### Subgroup analysis

Previous evidence suggested that gender, symptom score, GOLD grade and history of frequent acute exacerbation might influence therapeutic effect (Bhatta et al., 2020; Helvaci et al., 2020; Zhou et al., 2021; Siegfried, 2022). Clinicians should consider these factors in the management of COPD. Therefore, in this study, subgroup analysis was performed in frequency of AECOPD and lung function according to gender, CAT score, mMRC scale, GOLD grade and history of frequent acute exacerbation. Subgroup analysis showed that CAT score and mMRC scale score could affect frequency of AECOPD. Meanwhile, subgroup analysis also showed that history of frequent acute exacerbation could affect lung function. In patients with a CAT score at 10 or more, SHGBZK significantly decreased acute exacerbation during week 25–52 and week 52 (*p* = 0.0317, *p* = 0.0159). In patients with a mMRC score of 2 or more, SHGBZK significantly decreased acute exacerbation during week 24, week 25–52 and week 52 (*p* = 0.0129, *p* = 0.0047, *p* = 0.0378). In patients with higher history of frequent acute exacerbation (>2), SHGBZK significantly improved FVC (*p* = 0.0398) and PEF (*p* = 0.0262) compared with placebo during week 52. The results are shown in Supplementary Tables S7–S16.

## Discussion

Preliminary studies suggested that SHGBZK showed its potential to relieve symptoms and improve quality of life in COPD patients (Yin et al., 2001). However, there was no evidence for randomized controlled trials of SHGBZK to provide robust support on its efficacy and safety. Therefore, we performed a prospective randomized, double-blind, placebo-controlled trial to investigate the effectiveness of SHGBZK on COPD. In this study, we found that SHGBZK was effective in lowering CAT scores and mMRC scale, improving 6MWD and alleviating TCM symptoms. More interestingly, after subgroup analysis, SHGBZK resulted in a lower frequency of acute exacerbation in patients who had a CAT score of at least 10 or a mMRC score of at least 2. SHGBZK also significantly improved lung function outcomes in patients with frequent AECOPD.

Acute exacerbation of COPD are important events that have significant adverse consequences for patients. It has been reported that the in patients admitted to intensive care units for AECOPD, their mortality rate was about 11%–24% (Li et al., 2012). Therefore, the reduction in the frequency of exacerbation is a major goal of COPD management and an important indicator for evaluating the treatment (Criner et al., 2015). However, attempts to decrease the frequency of acute exacerbation with SHGBZK have not been fully successful. Although the frequency of acute exacerbation in SHGBZK group significantly reduced in the intervention stage and the follow-up stage, no significant between-group differences were observed. Then, subgroup analysis was conducted to investigate further explanations. Interestingly, we found that the frequency of acute exacerbation was significantly reduced in patients with a mMRC score of at least 2 or a CAT score of at least 10 by subgroup analysis. A possible explanation for this finding is that higher CAT and mMRC scores are associated with increased systemic inflammation and the inflammation is aggravated during acute exacerbation (Tu et al., 2014). Therefore, with the recovery of symptoms as measured by CAT and mMRC, the frequency of acute exacerbation decreased corresponding to a reduction in systemic inflammation (Hurst et al., 2010). This hypothesis is consistent with previous studies indicating that SHGBZK can reduce inflammatory cell infiltration, and thus promote inflammatory damage repair (Tang et al., 2018).

An accelerated decline in lung function is the main hallmark of COPD (Dransfield et al., 2017). Thus, lung function can be used not only to diagnose COPD, but also to evaluate clinical treatment effects. Our findings suggested that SHGBZK showed no significant effect on lung function. However, it is worth noting that SHGBZK significantly improved lung function in patients with frequent exacerbation. Previous studies suggested that airway inflammation was more prevalent in patients with frequent exacerbation, resulting in decline in lung function (Austin et al., 2016; Glynos et al., 2018). The drug reaction of SHGBZK on lung function may be influenced by inflammation status, but more studies are needed to verify this finding.

Symptoms, exercise tolerance, and health status were also observed in this study. Although COPD is defined by airflow limitation, the decision to seek medical help in practice is usually determined by the effect of symptoms on patients’ daily life (Wang et al., 2014). CAT and mMRC scale are two important instruments to assess efficacy of treatments in COPD patients and are strongly correlated with each other (Cheng et al., 2019; Pisi et al., 2021). Our findings revealed that SHGBZK were effective in improving both CAT and mMRC scores at the same time points (week 24), providing robust and reliable evidence on the efficacy of SHGBZK in improving health status and relieving symptoms. SHGBZK contains *Astragalus propinquus* (Huang Qi), *Polygonatum sibiricum* (Huang Jing), and *Schisandra chinensis* (Wu Wei Zi), all of which strengthen lung and spleen Qi. Meanwhile, SHGBZK improved TCM symptom score, supporting the hypothesis that SHGBZK can produce the effects of reinforcing lung and spleen Qi in the treatment of patients with stable COPD. Moreover, SHGBZK also significantly improved exercise tolerance, assessed by 6MWD, during week 52. It is interesting to note that the significant improvements in health status and symptoms were observed along with or before those in exercise tolerance and frequency of acute exacerbation. A potential explanation might be that SHGBZK accelerated the relief of symptoms, and thus led to an improvement in exercise tolerance and a reduction of acute exacerbation.

Our study has some limitations. First, this trial was conducted during the COVID-19 pandemic; although no COVID-19 patients were included in our trial, COVID-19 might still impact some of the outcomes. For example, COVID-19 lockdown was associated with a reduction in COPD exacerbation (González et al., 2021), and thus, in our study, the frequency of acute exacerbation was lower in both the treatment and placebo groups than the international standard. Second, the data of inflammatory cytokines were lacked in our study, making it difficult in analyzing the anti-inflammatory effect of SHGBZK. Third, there were only 98 participants included in our trials. The sample size might be inadequate to show significant effect in some outcomes (frequency of acute exacerbation, lung function, etc*.*), and some subgroups had small sample sizes, which might limit the reliability of the results.

## Conclusion

In summary, this trial showed that SHGBZK treatment for COPD was both effective and safe. SHGBZK significantly improved symptoms, exercise tolerance and health status of COPD patients. Moreover, subgroup analysis indicated SHGBZK had the potential to reduce frequency of exacerbation in patient with CAT scores of at least 10 or mMRC scores of at least 2 and to improve lung function in patients with frequent exacerbation. However, our findings should be considered preliminary and exploratory due to the limited sample size, and more high-quality clinical trials are needed to verify them.

## Data Availability

The original contributions presented in the study are included in the article/Supplementary Material, further inquiries can be directed to the corresponding author.

## References

[B1] Austin V. Crack P. J. Bozinovski S. Miller A. A. Vlahos R. (2016). COPD and stroke: Are systemic inflammation and oxidative stress the missing links? Clin. Sci. Lond. Engl. 1979) 130 (13), 1039–1050. 10.1042/CS20160043 PMC4876483 27215677

[B2] Bhatta L. Leivseth L. Mai X. M. Henriksen A. H. Carslake D. Chen Y. (2020). GOLD classifications, COPD hospitalization, and all-cause mortality in chronic obstructive pulmonary disease: The HUNT study. Int. J. Chron. Obstruct Pulmon Dis. 15, 225–233. 10.2147/COPD.S228958 32099347 PMC6999582

[B3] Cheng S. L. Lin C. H. Wang C. C. Chan M. C. Hsu J. Y. Hang L. W. (2019). Comparison between COPD Assessment Test (CAT) and modified Medical Research Council (mMRC) dyspnea scores for evaluation of clinical symptoms, comorbidities and medical resources utilization in COPD patients. J. Formos. Med. Assoc. = Taiwan yi zhi. 118 (13), 429–435. 10.1016/j.jfma.2018.06.018 30150099

[B4] Christiansen C. F. Løkke A. Bregnballe V. Prior T. S. Farver-Vestergaard I. (2023). COPD-related anxiety: A systematic review of patient perspectives. Int. J. Chron. Obstruct Pulmon Dis. 18, 1031–1046. 10.2147/COPD.S404701 37304765 PMC10257401

[B5] Chronic obstructive pulmonary disease group, Society of Respiratory Diseases, Chinese Medical Association (2013). Guidelines for the diagnosis and treatment of chronic obstructive pulmonary disease (revised version in 2013). Chin. J. Tuberc. Respir. 36 (4), 255–264. 10.3760/cma.j.issn.10010939.2013.04.007

[B6] Contoli M. Baraldo S. Conti V. Gnesini G. Marku B. Casolari P. (2020). Airway inflammatory profile is correlated with symptoms in stable COPD: A longitudinal proof-of-concept cohort study. Respirology 25 (1), 80–88. 10.1111/resp.13607 31251440

[B7] Criner G. J. Bourbeau J. Diekemper R. L. Ouellette D. R. Goodridge D. Hernandez P. (2015). Executive summary: Prevention of acute exacerbation of COPD: American college of chest physicians and Canadian thoracic society guideline. Chest 147 (4), 883–893. 10.1378/chest.14-1677 25320966 PMC4388123

[B8] Dransfield M. T. Kunisaki K. M. Strand M. J. Anzueto A. Bhatt S. P. Bowler R. P. (2017). Acute exacerbations and lung function loss in smokers with and without chronic obstructive pulmonary disease. Am. J. Respir. Crit. care Med. 195 (3), 324–330. 10.1164/rccm.201605-1014OC 27556408 PMC5328181

[B9] GBD 2015 Chronic Respiratory Disease Collaborators (2017). Global, regional, and national deaths, prevalence, disability-adjusted life years, and years lived with disability for chronic obstructive pulmonary disease and asthma, 1990-2015: A systematic analysis for the global burden of disease study 2015. Lancet Respir. Med. 5 (9), 691–706. 10.1016/S2213-2600(17)30293-X 28822787 PMC5573769

[B10] Glynos C. Bibli S. I. Katsaounou P. Pavlidou A. Magkou C. Karavana V. (2018). Comparison of the effects of e-cigarette vapor with cigarette smoke on lung function and inflammation in mice. Am. J. Physiol. Lung Cell. Mol. Physiol. 315 (5), L662–l72. 10.1152/ajplung.00389.2017 30091379

[B11] González J. Moncusí-Moix A. Benitez I. D. Santisteve S. Monge A. Fontiveros M. A. (2021). Clinical consequences of COVID-19 lockdown in patients with COPD: Results of a pre-post study in Spain. Chest 160 (1), 135–138. 10.1016/j.chest.2020.12.057 33444614 PMC7797779

[B12] Helvaci A. Izgu N. Ozdemir L. (2020). Relationship between symptom burden, medication adherence and spiritual well-being in patients with chronic obstructive pulmonary disease. J. Clin. Nurs. 29 (13-14), 2388–2396. 10.1111/jocn.15251 32221991

[B13] Hu Y. L. Chan X. M. liu S. Y. (2020). Study on the mechanism of Radix Astragali-Atractylodes macrocephala in the treatment of chronic obstructive pulmonary disease combined with transcriptome and network pharmacology. Chin. J. Integr. Med. 40 (10), 1196–1201.

[B14] Hurst J. R. V. J. Anzueto A. Locantore N. Müllerova H. Tal-Singer R. Miller B. (2010). Evaluation of COPD longitudinally to identify predictive surrogate endpoints (ECLIPSE) investigators. Susceptibility to exacerbation in chronic obstructive pulmonary disease. N. Engl. J. Med. 363 (12), 11. 10.1056/NEJMoa0909883 20843247

[B15] Li S. Y. Li J. S. Wang M. H. Xie Y. Yu X. Q. Sun Z. K. (2012). Effects of comprehensive therapy based on traditional Chinese medicine patterns in stable chronic obstructive pulmonary disease: A four-center, open-label, randomized, controlled study. BMC complementary Altern. Med. 12, 197. 10.1186/1472-6882-12-197 PMC3528455 23107470

[B16] Li Y. Xiong C. Zeng Y. Wei H. Zhuang G. Zhao L. (2019). Acupuncture treatment of lung-spleen Qi deficiency in stable chronic obstructive pulmonary disease: A randomized, open-label, controlled trial. J. Tradit. Chin. Med. 39 (6), 885–891. 10.19852/j.cnki.jtcm.2019.06.016 32186160

[B17] Liu Y. Xie X. Wang W. Zhao K. Xiao W. Xiao J. (2020). A randomized controlled trial for the effect of modified shenling baizhu powder on delaying the illness progress of COPD stable phase patients (GOLD 1-2 stages): A study protocol. Med. Baltim. 99 (43), 22700. 10.1097/MD.0000000000022700 PMC7581099 33120762

[B18] Mannino D. M. Higuchi K. Yu T. C. Zhou H. Li Y. Tian H. (2015). Economic burden of COPD in the presence of comorbidities. Chest 148 (1), 138–150. 10.1378/chest.14-2434 25675282 PMC4493870

[B19] Pisi R. Aiello M. Calzetta L. Frizzelli A. Tzani P. Bertorelli G. (2021). The COPD assessment test and the modified Medical Research Council scale are not equivalent when related to the maximal exercise capacity in COPD patients. Pulmonology 29, 194–199. 10.1016/j.pulmoe.2021.06.001 34233862

[B20] Professional Committee of Pulmonary Diseases (2012). Internal medicine branch, Chinese medical association. Guidelines for TCM diagnosis and treatment of chronic obstructive pulmonary disease (2011 edition). J. Tradit. Chin. Med. 53 (1), 80–84. 10.13288/j.11-2166/r.2012.01.011

[B21] Ritchie A. I. Brill S. E. Vlies B. H. Finney L. J. Allinson J. P. Alves-Moreira L. (2020). Targeted retreatment of incompletely recovered chronic obstructive pulmonary disease exacerbations with ciprofloxacin. A double-blind, randomized, placebo-controlled, multicenter, phase III clinical trial. Am. J. Respir. Crit. care Med. 202 (4), 549–557. 10.1164/rccm.201910-2058OC 32267724 PMC7427375

[B22] Schulz K. F. Altman D. G. Moher D., and CONSORT Group (2010). Consort 2010 statement: Updated guidelines for reporting parallel group randomized trials. Ann. Intern Med. 152, 1063–1070. 10.1097/AOG.0b013e3181d9d421 20410783

[B23] Siegfried J. M. (2022). Sex and gender differences in lung cancer and chronic obstructive lung disease. Endocrinology 163 (2), 254. 10.1210/endocr/bqab254 34927202

[B24] Tang S. Jing Y. Cao N. (2018). Study on the mechanism of Mucosal epithelium trauma of chronic obstructive pulmonary disease treated with Chinese herbal formula Gu-Ben Zhi-Ke. J. Basic Chin. Med. 24 (7), 1008–1011. 10.19945/j.cnki.issn.10063250.2018.07.044

[B25] Tu Y. H. Zhang Y. Fei G. H. (2014). Utility of the CAT in the therapy assessment of COPD exacerbations in China. BMC Pulm. Med. 14, 42. 10.1186/1471-2466-14-42 24618290 PMC3995795

[B26] Vogelmeier C. F. Criner G. J. Martinez F. J. Anzueto A. Barnes P. J. Bourbeau J. (2017). Global strategy for the diagnosis, management, and prevention of chronic obstructive lung disease 2017 Report: GOLD executive summary. Eur. Respir. J. 49 (3), 1700214. 10.1183/13993003.00214-2017 28182564

[B27] Wang C. Xu J. Yang L. Xu Y. Zhang X. Bai C. (2018). Prevalence and risk factors of chronic obstructive pulmonary disease in China (the China pulmonary health [CPH] study): A national cross-sectional study. Lancet (London, Engl. 391 (10131), 1706–1717. 10.1016/S0140-6736(18)30841-9 29650248

[B28] Wang M. Li J. Li S. Xie Y. (2014). Effects of comprehensive therapy based on traditional Chinese medicine patterns on older patients with chronic obstructive pulmonary disease: A subgroup analysis from a four-center, randomized, controlled study. Front. Med. 8 (3), 368–375. 10.1007/s11684-014-0360-0 25204290

[B29] Wang Y. Q. Liao Q. Tang S. H. Cai Z. Zhang H. C. (2020). Gubenzhike recipe ameliorates respiratory mucosal immunity in mice with chronic obstructive pulmonary disease through upregulation of the γδT lymphocytes and KGF levels. Evid. Based Complement. Altern. Med. 2020, 3056797. 10.1155/2020/3056797 PMC7128036 32280354

[B30] Yin Y. Zhang H. Chao E. (2001). Clinical study on delayed treatment of chronic bronchitis with Guben Zhike Capsule. J. Beijing Univ. Tradit. Chin. Med. 24 (2), 58–61.

[B31] Zhou L. Fang Y. Liu W. Zhang J. Wang Y. Xie S. (2021). Comparison of immediate and sequential withdrawal of a systemic glucocorticoid in the treatment of acute exacerbations of chronic obstructive pulmonary disease: A multicenter, randomized, double-blind, parallel-controlled, open-label study. Front. Mol. Biosci. 8, 639079. 10.3389/fmolb.2021.639079 34095219 PMC8173198

[B32] Zu Y. Li D. Lei X. Zhang H. (2019). Effects of the Chinese herbal formula san-huang gu-ben zhi-ke treatment on stable chronic obstructive pulmonary disease: Study protocol of a randomized, double-blind, placebo-controlled trial. Trials 20 (1), 647. 10.1186/s13063-019-3729-1 31775843 PMC6880401

