# Supplementary material for: Effects of the Chinese herbal formula San-Huang Gu-Ben Zhi-Ke treatment on stable chronic obstructive pulmonary disease: a randomized, double-blind, placebo-controlled trial
Source: Front Pharmacol. 2023 Jun 27;14:1164818. doi: 10.3389/fphar.2023.1164818 (PMC10335626; doi:10.3389/fphar.2023.1164818)
Supplement: Supplementary file 1 [file Table1.DOCX]

**Table S1**

Main components of the SHGBZK Chinese medicine

| Chinese name | Latin name | Amount (g) |
| --- | --- | --- |
| Huang Qi | Astragalus propinquus | 15 |
| Huang Jing | Polygonatum sibiricum | 12 |
| Chen Pi | Pericarpium citri reticulatae | 10 |
| Bai Bu | Stemona japonica | 10 |
| Wu Wei Zi | Schisandra chinensis | 8 |
| Chi Shao | Paeonia lactiflora Pall | 10 |
| Huang Qin | Scutellaria baicalensis Georgi | 8 |

**Table S2**

TCM symptom score

| Main symptom (score) | Normal (0) | Light (2) | Medium (4) | Severe (6) |
| --- | --- | --- | --- | --- |
| Cough | No | Intermittent cough during the day | Cough during day and night without affecting work and sleep | Cough frequently during day and night which affects work and sleep |
| Sputum | No | A small amount of sputum | Sputum and wheezy phlegm | A large amount of sputum and loud wheezy phlegm |
| Shortness of breath | No | Shortness of breath after work | Fatigue and shortness of breath | Short of breath when quiet |
| Secondary symptoms | Normal (0) | Light (1) | Medium (2) | Severe (3) |
| Spontaneous  perspiration | No | Sweat while eating | Intermittent sweat | Sweat soaks clothes, sweat more after work |
| Loss of appetite | No | Loss of appetite, but eat as usual | Eat less but not under one-third of usual | Eat less than one-third of usual |
| Weak | No | Can do light physical work | Can’t do physical work | General fatigue, intend to stay in bed |
| Abdominal distention  and loose stool | No | Light abdominal distention and the  stool is not forming | Abdominal distention is obvious and loose stool | Abdominal distention is obvious and water-like stool |

**Table S3**

Difference of BODE between the two groups at different time points

| Follow-up | SHGBZK Chinese medicine | | Placebo | | P Value |
| --- | --- | --- | --- | --- | --- |
|  | N | Mean(SD) | N | Mean(SD) |  |
| T0 | 42(8) | 3.50(1.77) | 43(5) | 3.35(1.76) | 0.6939 |
| T1 | 42(8) | 3.05(1.48) | 44(4) | 3.20(1.69) | 0.6490 |
| T1-T0 | 43(7) | -0.45(2.18) | 43(5) | 0.16(1.61) | 0.1446 |
| T2 | 35(15) | 3.23(1.37) | 29(19) | 3.55(1.88) | 0.4309 |
| T2-T0 | 30(20) | -0.17(1.21) | 32(16) | -0.01(1.98) | 0.7052 |

Note. Data are shown as mean±SD. P values were calculated by t-test, signed rank sum test or chi-square test. * p< 0.05. T0: baseline, T1: week 24, T2: week 52.

**Table S4**

Difference of TCM symptoms between the two groups at different time points

| Follow-up | SHGBZK Chinese medicine | | Placebo | | P Value |
| --- | --- | --- | --- | --- | --- |
|  | N | Mean(SD) | N | Mean(SD) |  |
| T0 | 50(0) | 10.22(3.98) | 48(0) | 9.29(4.38) | 0.2744 |
| T1 | 50(0) | 8.92(4.38) | 48(0) | 7.77(3.54) | 0.1576 |
| T1-T0 | 50(0) | -1.30(4.50) | 48(0) | -1.52(3.33) | 0.7837 |
| T2 | 50(0) | 7.76(3.59) | 48(0) | 8.02(4.28) | 0.7442 |
| T2-T0 | 50(0) | -2.46(4.58) | 48(0) | -1.27(3.71) | 0.1620 |
| T3 | 50(0) | 7.44(3.75) | 48(0) | 8.04(3.63) | 0.4219 |
| T3-T0 | 50(0) | -2.78(4.99) | 48(0) | -1.25(4.34) | 0.1092 |
| T4 | 50(0) | 7.20(3.34) | 48(0) | 7.65(3.54) | 0.5226 |
| T4-T0 | 50(0) | -3.02(4.56) | 48(0) | -1.65(3.63) | 0.1032 |
| T5 | 50(0) | 7.14(3.25) | 48(0) | 8.40(4.06) | 0.0932 |
| T5-T0 | 50(0) | -3.08(4.81) | 48(0) | -0.90(4.40) | **0.0212*** |
| T6 | 50(0) | 6.98(3.46) | 48(0) | 8.38(3.47) | **0.0491*** |
| T6-T0 | 50(0) | -3.24(4.84) | 48(0) | -0.92(3.80) | **0.0098*** |
| T7 | 50(0) | 6.90(4.27) | 48(0) | 7.13(3.65) | 0.7801 |
| T7-T0 | 50(0) | -3.32(5.61) | 48(0) | -2.17(4.28) | 0.2572 |
| T8 | 50(0) | 6.62(4.33) | 48(0) | 6.90(3.36) | 0.7262 |
| T8-T0 | 50(0) | -3.60(5.59) | 48(0) | -2.40(3.36) | 0.2018 |
| T9 | 50(0) | 6.92(4.10) | 48(0) | 6.98(4.10) | 0.9432 |
| T9-T0 | 50(0) | -3.30(4.99) | 48(0) | -2.31(3.92) | 0.2801 |

Note. Data are shown mean±SD. P values were calculated by t-test, signed rank sum test or chi-square test. * p< 0.05. T0: baseline, T1: week 4, T2: week 8, T3: week 12, T4: week 16, T5: week 20, T6: week 24, T7: week 32, T8: week 40, T9: week 52.

**Table S5**

Difference of concomitant medications between the two groups

| Variable | SHGBZK Chinese medicine Group (N=50) | Placebo Group  (N=48) | P Value |
| --- | --- | --- | --- |
| Whether to use concomitant medication or not |  |  | 0.1907 |
| concomitant medication used (%) | 46(92.00) | 40(83.33) |  |
| No concomitant medication used (%) | 4( 8.00) | 8(16.67) |  |

Note. Data are shown n (%). P values were calculated by t-test, signed rank sum test or chi-square test. * p< 0.05.

**Table S6**

Incidence rate of adverse events

| Variable | SHGBZK Chinese medicine Group (N=50) | Placebo Group  (N=48) | P Value |
| --- | --- | --- | --- |
| Adverse event | 22(44.00) | 21(43.75) | 0.9801 |
| Adverse reaction | 14(28.00) | 17(35.42) | 0.4300 |
| Severe adverse event | 1( 2.00) | 0( 0.00) | 1.0000 |
| Severe adverse reaction | 0( 0.00) | 0( 0.00) | 1.0000 |
| adverse event to drop | 0( 0.00) | 1( 2.08) | 0.9836 |
| adverse reaction to drop | 0( 0.00) | 1( 2.08) | 0.9836 |

Note. Data are shown n (%). P values were calculated by t-test, signed rank sum test or chi-square test. * p< 0.05.

**Table S7**

Lung function between two groups at different time points in subgroup stratified by CAT scores

| Variable | CAT <10 | | | | | CAT ≥10 | | | | |
| --- | --- | --- | --- | --- | --- | --- | --- | --- | --- | --- |
|  | SHGBZK | | Placebo | | P values | SHGBZK | | Placebo | | P values |
|  | N | Mean (SD) | N | Mean (SD) |  | N | Mean (SD) | N | Mean (SD) |  |
| FEV1 |  |  |  |  |  |  |  |  |  |  |
| T0 | 23(3) | 1.59(0.65) | 20(1) | 1.92(0.59) | 0.0919 | 21(3) | 1.35(0.64) | 27(0) | 1.53(0.77) | 0.4001 |
| T1 | 25(1) | 1.66(0.71) | 21(0) | 1.88(0.56) | 0.1226 | 23(1) | 1.30(0.62) | 27(0) | 1.56(0.72) | 0.1797 |
| T2 | 25(1) | 1.76(0.82) | 21(0) | 1.78(0.56) | 0.6043 | 23(1) | 1.24(0.61) | 27(0) | 1.49(0.72) | 0.1952 |
| T1-T0 | 23(3) | -0.04(0.35) | 20(1) | -0.07(0.22) | 0.8738 | 21(3) | -0.01(0.12) | 27(0) | 0.03(0.24) | 0.9831 |
| T2-T0 | 23(3) | 0.07(0.62) | 20(1) | -0.17(0.20) | 0.0946 | 21(3) | -0.06(0.20) | 27(0) | -0.03(0.15) | 0.7773 |
| FVC |  |  |  |  |  |  |  |  |  |  |
| T0 | 23(3) | 2.99(0.90) | 20(1) | 3.29(0.87) | 0.2676 | 21(3) | 2.73(0.72) | 27(0) | 3.11(0.96) | 0.1333 |
| T1 | 25(1) | 3.16(0.90) | 21(0) | 3.23(0.80) | 0.7820 | 23(1) | 2.64(0.78) | 27(0) | 3.10(0.96) | 0.0703 |
| T2 | 25(1) | 3.14(0.98) | 21(0) | 3.15(0.86) | 0.9531 | 23(1) | 2.49(0.72) | 27(0) | 3.02(0.96) | 0.0332 |
| T1-T0 | 23(3) | 0.04(0.38) | 20(1) | -0.09(0.33) | 0.2419 | 21(3) | -0.12(0.31) | 27(0) | -0.01(0.30) | 0.4861 |
| T2-T0 | 23(3) | 0.02(0.43) | 20(1) | -0.17(0.34) | 0.1206 | 21(3) | -0.21(0.33) | 27(0) | -0.09(0.36) | 0.6449 |
| FEV1% |  |  |  |  |  |  |  |  |  |  |
| T0 | 23(3) | 52.91(13.62) | 20(1) | 58.17(9.19) | 0.1514 | 21(3) | 48.15(15.07) | 27(0) | 47.33(13.37) | 0.8437 |
| T1 | 25(1) | 51.67(12.02) | 21(0) | 58.18(9.12) | 0.0916 | 23(1) | 47.75(14.98) | 27(0) | 49.40(13.87) | 0.6882 |
| T2 | 25(1) | 54.46(11.99) | 21(0) | 56.79(9.97) | 0.4825 | 23(1) | 47.98(13.99) | 27(0) | 47.95(13.36) | 0.9930 |
| T1-T0 | 23(3) | -2.23(8.70) | 20(1) | -0.42(3.96) | 0.1921 | 21(3) | 1.55(5.25) | 27(0) | 2.06(11.03) | 0.3161 |
| T2-T0 | 23(3) | 0.81(13.86) | 20(1) | -1.88(5.18) | 0.6964 | 21(3) | 1.21(6.09) | 27(0) | 0.62(3.25) | 0.6380 |
| PEF |  |  |  |  |  |  |  |  |  |  |
| T0 | 23(3) | 4.26(1.82) | 20(1) | 5.57(1.80) | 0.0223 | 21(3) | 3.94(1.84) | 27(0) | 4.20(2.03) | 0.6515 |
| T1 | 25(1) | 4.77(2.13) | 21(0) | 5.56(1.93) | 0.1366 | 23(1) | 4.01(1.79) | 27(0) | 4.38(1.93) | 0.4851 |
| T2 | 25(1) | 5.08(2.47) | 21(0) | 5.43(1.97) | 0.3104 | 23(1) | 3.95(1.83) | 27(0) | 4.26(2.10) | 0.7043 |
| T1-T0 | 23(3) | 0.23(1.07) | 20(1) | 0.03(0.85) | 0.7979 | 21(3) | 0.08(0.50) | 27(0) | 0.18(0.92) | 0.7750 |
| T2-T0 | 23(3) | 0.53(1.75) | 20(1) | -0.11(0.99) | 0.4720 | 21(3) | 0.00(0.70) | 27(0) | 0.06(0.47) | 0.7144 |
| MMEF |  |  |  |  |  |  |  |  |  |  |
| T0 | 23(3) | 0.80(0.64) | 20(1) | 0.85(0.46) | 0.2375 | 21(3) | 0.57(0.42) | 27(0) | 0.61(0.47) | 0.9420 |
| T1 | 25(1) | 0.68(0.45) | 21(0) | 0.83(0.42) | 0.1199 | 23(1) | 0.52(0.41) | 27(0) | 0.65(0.47) | 0.2886 |
| T2 | 25(1) | 0.82(0.74) | 21(0) | 0.76(0.44) | 0.7490 | 23(1) | 0.52(0.38) | 27(0) | 0.59(0.46) | 0.6754 |
| T1-T0 | 23(3) | -0.18(0.59) | 20(1) | -0.05(0.24) | 0.3670 | 21(3) | -0.02(0.19) | 27(0) | 0.04(0.32) | 0.6538 |
| T2-T0 | 23(3) | -0.02(0.95) | 20(1) | -0.12(0.22) | 0.3355 | 21(3) | -0.03(0.17) | 27(0) | -0.02(0.12) | 0.8423 |

Note. Data are shown as mean±SD. P values were calculated by t-test, signed rank sum test or chi-square test. * p< 0.05. T0: baseline, T1: week 24, T2: week 52.

**Table S8**

Frequency of acute exacerbations between two groups at different time points in subgroup stratified by CAT scores

| Follow-up | CAT <10 | | | | | CAT ≥10 | | | | |
| --- | --- | --- | --- | --- | --- | --- | --- | --- | --- | --- |
|  | SHGBZK | | Placebo | | P values | SHGBZK | | Placebo | | P values |
|  | N | Mean (SD) | N | Mean (SD) |  | N | Mean (SD) | N | Mean (SD) |  |
| Baseline | 25(1) | 1.72(1.10) | 21(0) | 1.95(2.04) | 1.0000 | 24(0) | 2.29(2.20) | 26(1) | 2.12(1.53) | 0.7025 |
| Week 24 | 26(0) | 0.03(0.08) | 21(0) | 0.02(0.05) | 0.8102 | 24(0) | 0.01(0.03) | 27(0) | 0.07(0.16) | 0.1054 |
| Week 52 | 26(0) | 0.15(0.46) | 21(0) | 0.10(0.30) | 0.8102 | 24(0) | 0.04(0.20) | 27(0) | 0.59(1.45) | **0.0317*** |
| Week 24-52 | 26(0) | 0.01(0.06) | 21(0) | 0.00(0.00) | 0.3920 | 24(0) | 0.00(0.00) | 27(0) | 0.06(0.12) | **0.0159*** |

Note. Data are shown as mean±SD. P values were calculated by t-test, signed rank sum test or chi-square test. * p< 0.05. T0: baseline, T1: week 24, T2: week 52.

**Table S9**

Lung function between two groups at different time points in subgroup stratified by mMRC scores

| Variable | mMRC < 2 | | | | | mMRC ≥ 2 | | | | |
| --- | --- | --- | --- | --- | --- | --- | --- | --- | --- | --- |
|  | SHGBZK | | Placebo | | P values | SHGBZK | | Placebo | | P values |
|  | N | Mean (SD) | N | Mean (SD) |  | N | Mean (SD) | N | Mean (SD) |  |
| FEV1 |  |  |  |  |  |  |  |  |  |  |
| T0 | 20(2) | 1.75(0.61) | 22(0) | 1.96(0.73) | 0.3275 | 24(4) | 1.25(0.60) | 25(1) | 1.46(0.64) | 0.2286 |
| T1 | 22(0) | 1.76(0.70) | 22(0) | 1.86(0.68) | 0.6360 | 26(2) | 1.25(0.60) | 26(0) | 1.56(0.64) | 0.0766 |
| T2 | 22(0) | 1.84(0.80) | 22(0) | 1.81(0.67) | 0.8985 | 26(2) | 1.23(0.62) | 26(0) | 1.46(0.63) | 0.1785 |
| T1-T0 | 20(2) | -0.10(0.15) | 22(0) | -0.09(0.21) | 0.8970 | 24(4) | 0.03(0.32) | 25(1) | 0.06(0.24) | 0.9419 |
| T2-T0 | 20(2) | -0.01(0.61) | 22(0) | -0.15(0.19) | 0.9097 | 24(4) | 0.03(0.32) | 25(1) | -0.04(0.16) | 0.1156 |
| FVC |  |  |  |  |  |  |  |  |  |  |
| T0 | 20(2) | 3.24(0.71) | 22(0) | 3.30(0.96) | 0.8093 | 24(4) | 2.55(0.79) | 25(1) | 3.09(0.89) | 0.0302 |
| T1 | 22(0) | 3.29(0.81) | 22(0) | 3.27(0.95) | 0.9351 | 26(2) | 2.59(0.80) | 26(0) | 3.06(0.84) | 0.0470 |
| T2 | 22(0) | 3.24(0.87) | 22(0) | 3.20(0.96) | 0.8623 | 26(2) | 2.48(0.81) | 26(0) | 2.98(0.87) | 0.0339 |
| T1-T0 | 20(2) | -0.08(0.38) | 22(0) | -0.03(0.36) | 0.6722 | 24(4) | 0.00(0.34) | 25(1) | -0.05(0.28) | 0.4478 |
| T2-T0 | 20(2) | -0.13(0.42) | 22(0) | -0.11(0.38) | 0.8575 | 24(4) | -0.05(0.39) | 25(1) | -0.13(0.33) | 0.1997 |
| FEV1% |  |  |  |  |  |  |  |  |  |  |
| T0 | 20(2) | 53.07(12.46) | 22(0) | 58.07(9.21) | 0.1439 | 24(4) | 48.61(15.75) | 25(1) | 46.55(13.33) | 0.6626 |
| T1 | 22(0) | 52.57(12.64) | 22(0) | 56.46(9.65) | 0.2577 | 26(2) | 47.44(14.03) | 26(0) | 50.51(14.42) | 0.4397 |
| T2 | 22(0) | 54.76(12.52) | 22(0) | 56.33(11.18) | 0.6622 | 26(2) | 48.47(13.42) | 26(0) | 47.99(12.81) | 0.8955 |
| T1-T0 | 20(2) | -1.54(5.47) | 22(0) | -1.61(3.80) | 0.9610 | 24(4) | 0.51(8.74) | 25(1) | 3.31(11.07) | 0.5657 |
| T2-T0 | 20(2) | 0.88(13.54) | 22(0) | -1.74(4.96) | 0.7056 | 24(4) | 1.11(8.04) | 25(1) | 0.69(3.36) | 0.7998 |
| PEF |  |  |  |  |  |  |  |  |  |  |
| T0 | 20(2) | 4.61(1.85) | 22(0) | 5.34(1.87) | 0.2123 | 24(4) | 3.69(1.71) | 25(1) | 4.29(2.09) | 0.2711 |
| T1 | 22(0) | 5.02(1.98) | 22(0) | 5.33(2.03) | 0.6130 | 26(2) | 3.88(1.88) | 26(0) | 4.53(1.93) | 0.1155 |
| T2 | 22(0) | 5.36(2.34) | 22(0) | 5.30(2.15) | 0.9267 | 26(2) | 3.84(1.92) | 26(0) | 4.33(2.00) | 0.3141 |
| T1-T0 | 20(2) | 0.11(0.73) | 22(0) | -0.01(0.81) | 0.6216 | 24(4) | 0.19(0.94) | 25(1) | 0.22(0.95) | 0.8448 |
| T2-T0 | 20(2) | 0.44(1.76) | 22(0) | -0.04(0.95) | 0.7721 | 24(4) | 0.14(0.95) | 25(1) | 0.01(0.50) | 0.7531 |
| MMEF |  |  |  |  |  |  |  |  |  |  |
| T0 | 20(2) | 0.77(0.41) | 22(0) | 0.91(0.54) | 0.5124 | 24(4) | 0.62(0.65) | 25(1) | 0.53(0.34) | 0.7640 |
| T1 | 22(0) | 0.76(0.47) | 22(0) | 0.80(0.47) | 0.5974 | 26(2) | 0.47(0.35) | 26(0) | 0.67(0.44) | 0.0886 |
| T2 | 22(0) | 0.89(0.76) | 22(0) | 0.79(0.51) | 0.7962 | 26(2) | 0.49(0.37) | 26(0) | 0.56(0.38) | 0.4419 |
| T1-T0 | 20(2) | -0.07(0.20) | 22(0) | -0.11(0.22) | 0.8106 | 24(4) | -0.13(0.59) | 25(1) | 0.11(0.31) | 0.1790 |
| T2-T0 | 20(2) | 0.08(0.81) | 22(0) | -0.12(0.22) | 0.6502 | 24(4) | -0.11(0.57) | 25(1) | -0.01(0.11) | 0.8233 |

Note. Data are shown as mean±SD. P values were calculated by t-test, signed rank sum test or chi-square test. * p< 0.05. T0: baseline, T1: week 24, T2: week 52.

**Table S10**

Frequency of acute exacerbations between two groups at different time points in subgroup stratified by mMRC scores

| Follow-up | mMRC < 2 | | | | | mMRC ≥ 2 | | | | |
| --- | --- | --- | --- | --- | --- | --- | --- | --- | --- | --- |
|  | SHGBZK | | Placebo | | P values | SHGBZK | | Placebo | | P values |
|  | N | Mean (SD) | N | Mean (SD) |  | N | Mean (SD) | N | Mean (SD) |  |
| Baseline | 22(0) | 1.77(1.38) | 22(0) | 2.27(2.19) | 0.5114 | 27(1) | 2.19(1.98) | 25(1) | 1.84(1.28) | 0.9320 |
| Week 24 | 22(0) | 0.02(0.06) | 22(0) | 0.00(0.00) | 0.0807 | 28(0) | 0.01(0.06) | 26(0) | 0.08(0.16) | **0.0192*** |
| Week 52 | 22(0) | 0.14(0.35) | 22(0) | 0.00(0.00) | 0.0807 | 28(0) | 0.07(0.38) | 26(0) | 0.69(1.46) | **0.0047*** |
| Week 24-52 | 22(0) | 0.00(0.00) | 22(0) | 0.00(0.00) | 1.0000 | 28(0) | 0.01(0.05) | 26(0) | 0.06(0.12) | **0.0378*** |

Note. Data are shown as mean±SD. P values were calculated by t-test, signed rank sum test or chi-square test. * p< 0.05. T0: baseline, T1: week 24, T2: week 52.

**Table S11**

Lung function between two groups at different time points in subgroup stratified by history of frequent acute exacerbations

| Variable | frequency of acute exacerbations ≤ 2 | | | | | frequency of acute exacerbations > 2 | | | | |
| --- | --- | --- | --- | --- | --- | --- | --- | --- | --- | --- |
|  | SHGBZK | | Placebo | | P values | SHGBZK | | Placebo | | P values |
|  | N | Mean (SD) | N | Mean (SD) |  | N | Mean (SD) | N | Mean (SD) |  |
| FEV1 |  |  |  |  |  |  |  |  |  |  |
| T0 | 32(5) | 1.46(0.66) | 38(1) | 1.76(0.72) | 0.0735 | 11(1) | 1.46(0.65) | 8(0) | 1.48(0.67) | 0.9407 |
| T1 | 36(1) | 1.48(0.71) | 39(0) | 1.78(0.65) | 0.0576 | 11(1) | 1.46(0.66) | 8(0) | 1.42(0.63) | 0.9010 |
| T2 | 36(1) | 1.45(0.74) | 39(0) | 1.70(0.65) | 0.0689 | 11(1) | 1.67(0.88) | 8(0) | 1.36(0.61) | 0.5087 |
| T1-T0 | 32(5) | -0.04(0.30) | 38(1) | -0.00(0.25) | 0.4812 | 11(1) | 0.00(0.09) | 8(0) | -0.06(0.20) | 0.4151 |
| T2-T0 | 32(5) | -0.06(0.34) | 38(1) | -0.09(0.19) | 0.8780 | 11(1) | 0.21(0.73) | 8(0) | -0.13(0.16) | 0.0536 |
| FVC |  |  |  |  |  |  |  |  |  |  |
| T0 | 32(5) | 2.81(0.82) | 38(1) | 3.31(0.90) | 0.0204 | 11(1) | 2.97(0.88) | 8(0) | 2.78(0.90) | 0.6473 |
| T1 | 36(1) | 2.89(0.88) | 39(0) | 3.28(0.87) | 0.0607 | 11(1) | 2.92(0.91) | 8(0) | 2.70(0.83) | 0.5900 |
| T2 | 36(1) | 2.78(0.95) | 39(0) | 3.22(0.89) | 0.0401 | 11(1) | 2.93(0.87) | 8(0) | 2.51(0.80) | 0.2962 |
| T1-T0 | 32(5) | -0.03(0.40) | 38(1) | -0.04(0.31) | 0.9901 | 11(1) | -0.05(0.24) | 8(0) | -0.08(0.36) | 0.8209 |
| T2-T0 | 32(5) | -0.11(0.46) | 38(1) | -0.09(0.36) | 0.8832 | 11(1) | -0.08(0.36) | 8(0) | -0.27(0.26) | **0.0398*** |
| FEV1% |  |  |  |  |  |  |  |  |  |  |
| T0 | 32(5) | 51.06(15.34) | 38(1) | 52.34(12.27) | 0.6991 | 11(1) | 48.63(12.07) | 8(0) | 52.79(14.84) | 0.5094 |
| T1 | 36(1) | 49.64(14.23) | 39(0) | 54.12(12.31) | 0.1477 | 11(1) | 49.45(11.91) | 8(0) | 51.83(13.27) | 0.6870 |
| T2 | 36(1) | 50.16(13.43) | 39(0) | 52.06(12.48) | 0.5281 | 11(1) | 54.55(13.15) | 8(0) | 53.34(12.77) | 0.8441 |
| T1-T0 | 32(5) | -0.86(8.54) | 38(1) | 1.45(9.42) | 0.3751 | 11(1) | 0.82(2.93) | 8(0) | -0.96(5.41) | 0.3671 |
| T2-T0 | 32(5) | -0.66(8.23) | 38(1) | -0.67(4.50) | 0.9943 | 11(1) | 5.92(15.86) | 8(0) | 0.55(3.72) | 0.4256 |
| PEF |  |  |  |  |  |  |  |  |  |  |
| T0 | 32(5) | 4.15(1.82) | 38(1) | 4.89(1.98) | 0.1097 | 11(1) | 3.97(1.95) | 8(0) | 4.65(2.25) | 0.4874 |
| T1 | 36(1) | 4.50(2.06) | 39(0) | 5.06(1.90) | 0.2233 | 11(1) | 4.10(1.89) | 8(0) | 4.49(2.30) | 0.6838 |
| T2 | 36(1) | 4.45(2.23) | 39(0) | 4.95(2.07) | 0.2032 | 11(1) | 4.86(2.43) | 8(0) | 4.29(2.14) | 0.6067 |
| T1-T0 | 32(5) | 0.17(0.98) | 38(1) | 0.18(0.97) | 0.3908 | 11(1) | 0.13(0.29) | 8(0) | -0.16(0.31) | 0.0546 |
| T2-T0 | 32(5) | 0.08(1.03) | 38(1) | 0.06(0.76) | 0.3451 | 11(1) | 0.89(2.05) | 8(0) | -0.36(0.56) | **0.0262*** |
| MMEF |  |  |  |  |  |  |  |  |  |  |
| T0 | 32(5) | 0.72(0.59) | 38(1) | 0.74(0.49) | 0.5015 | 11(1) | 0.60(0.45) | 8(0) | 0.64(0.45) | 1.0000 |
| T1 | 36(1) | 0.60(0.43) | 39(0) | 0.78(0.47) | 0.0309 | 11(1) | 0.61(0.46) | 8(0) | 0.55(0.34) | 0.9014 |
| T2 | 36(1) | 0.60(0.40) | 39(0) | 0.70(0.47) | 0.3616 | 11(1) | 0.90(1.05) | 8(0) | 0.55(0.38) | 0.5355 |
| T1-T0 | 32(5) | -0.14(0.52) | 38(1) | 0.03(0.30) | 0.2130 | 11(1) | 0.01(0.04) | 8(0) | -0.09(0.24) | 0.4254 |
| T2-T0 | 32(5) | -0.14(0.51) | 38(1) | -0.06(0.19) | 0.9717 | 11(1) | 0.30(1.03) | 8(0) | -0.09(0.14) | 0.1664 |

Note. Data are shown as mean±SD. P values were calculated by t-test, signed rank sum test or chi-square test. * p< 0.05. T0: baseline, T1: week 24, T2: week 52.

**Table S12**

Frequency of acute exacerbations between two groups at different time points in subgroup stratified by history of frequent acute exacerbations

| Follow-up | frequency of acute exacerbations ≤ 2 | | | | | frequency of acute exacerbations > 2 | | | | |
| --- | --- | --- | --- | --- | --- | --- | --- | --- | --- | --- |
|  | SHGBZK | | Placebo | | P values | SHGBZK | | Placebo | | P values |
|  | N | Mean (SD) | N | Mean (SD) |  | N | Mean (SD) | N | Mean (SD) |  |
| Baseline | 37(0) | 1.24(0.72) | 39(0) | 1.46(0.76) | 0.1370 | 12(0) | 4.33(1.87) | 8(0) | 4.88(2.47) | 0.6544 |
| Week 24 | 37(0) | 0.02(0.07) | 39(0) | 0.05(0.14) | 0.3038 | 12(0) | 0.01(0.05) | 8(0) | 0.02(0.06) | 0.8240 |
| Week 52 | 37(0) | 0.11(0.39) | 39(0) | 0.41(1.23) | 0.1933 | 12(0) | 0.08(0.29) | 8(0) | 0.25(0.46) | 0.3501 |
| Week 24-52 | 37(0) | 0.01(0.05) | 39(0) | 0.04(0.10) | 0.5415 | 12(0) | 0.00(0.00) | 8(0) | 0.02(0.05) | 0.2616 |

Note. Data are shown as mean±SD. P values were calculated by t-test, signed rank sum test or chi-square test. * p< 0.05. T0: baseline, T1: week 24, T2: week 52.

**Table S13**

Lung function between two groups at different time points in subgroup stratified by GOLD grade

| Variable | GOLD 1-2 | | | | | GOLD 3-4 | | | | |
| --- | --- | --- | --- | --- | --- | --- | --- | --- | --- | --- |
|  | SHGBZK | | Placebo | | P values | SHGBZK | | Placebo | | P values |
|  | N | Mean (SD) | N | Mean (SD) |  | N | Mean (SD) | N | Mean (SD) |  |
| FEV1 |  |  |  |  |  |  |  |  |  |  |
| T0 | 27(3) | 1.83(0.56) | 30(1) | 2.06(0.58) | 0.1297 | 17(3) | 0.91(0.28) | 17(0) | 1.05(0.43) | 0.2961 |
| T1 | 29(1) | 1.83(0.60) | 31(0) | 2.00(0.56) | 0.1332 | 19(1) | 0.96(0.44) | 17(0) | 1.13(0.46) | 0.2410 |
| T2 | 29(1) | 1.90(0.68) | 31(0) | 1.93(0.55) | 0.5009 | 19(1) | 0.92(0.44) | 17(0) | 1.05(0.46) | 0.3663 |
| T1-T0 | 27(3) | -0.08(0.19) | 30(1) | -0.07(0.19) | 0.7535 | 17(3) | 0.06(0.34) | 17(0) | -0.09(0.28) | 0.3642 |
| T2-T0 | 27(3) | 0.00(0.53) | 30(1) | -0.15(0.19) | 0.1782 | 17(3) | 0.02(0.36) | 17(0) | 0.01(0.13) | 0.8347 |
| FVC |  |  |  |  |  |  |  |  |  |  |
| T0 | 27(3) | 3.15(0.76) | 30(1) | 3.49(0.77) | 0.1025 | 17(3) | 2.41(0.72) | 17(0) | 2.66(0.94) | 0.5127 |
| T1 | 29(1) | 3.20(0.78) | 31(0) | 3.44(0.75) | 0.1599 | 19(1) | 2.47(0.84) | 17(0) | 2.64(0.91) | 0.6458 |
| T2 | 29(1) | 3.18(0.82) | 31(0) | 3.34(0.76) | 0.4312 | 19(1) | 2.29(0.79) | 17(0) | 2.60(0.98) | 0.3334 |
| T1-T0 | 27(3) | -0.06(0.32) | 30(1) | -0.05(0.33) | 0.9547 | 17(3) | 0.00(0.41) | 17(0) | -0.02(0.29) | 0.8745 |
| T2-T0 | 27(3) | -0.07(0.36) | 30(1) | -0.16(0.36) | 0.0909 | 17(3) | -0.11(0.47) | 17(0) | -0.06(0.33) | 0.7060 |
| FEV1% |  |  |  |  |  |  |  |  |  |  |
| T0 | 27(3) | 58.26(11.88) | 30(1) | 58.90(8.46) | 0.8154 | 17(3) | 38.53(8.44) | 17(0) | 39.68(9.74) | 0.7153 |
| T1 | 29(1) | 56.83(9.92) | 31(0) | 58.30(8.80) | 0.5478 | 19(1) | 39.04(11.06) | 17(0) | 44.02(13.71) | 0.2370 |
| T2 | 29(1) | 58.93(9.40) | 31(0) | 57.92(9.76) | 0.6838 | 19(1) | 39.79(9.36) | 17(0) | 40.68(9.48) | 0.7768 |
| T1-T0 | 27(3) | -1.89(7.41) | 30(1) | -0.88(3.64) | 0.6646 | 17(3) | 1.90(7.05) | 17(0) | 4.34(13.35) | 0.7412 |
| T2-T0 | 27(3) | 0.38(12.58) | 30(1) | -1.27(4.62) | 0.6481 | 17(3) | 2.00(7.19) | 17(0) | 1.01(3.38) | 0.9309 |
| PEF |  |  |  |  |  |  |  |  |  |  |
| T0 | 27(3) | 4.88(1.85) | 30(1) | 5.78(1.76) | 0.0652 | 17(3) | 2.87(0.80) | 17(0) | 3.02(1.09) | 0.6539 |
| T1 | 29(1) | 5.23(1.83) | 31(0) | 5.72(1.86) | 0.3148 | 19(1) | 3.13(1.51) | 17(0) | 3.40(1.26) | 0.1832 |
| T2 | 29(1) | 5.47(2.13) | 31(0) | 5.70(1.95) | 0.5943 | 19(1) | 3.12(1.58) | 17(0) | 3.08(1.09) | 0.5792 |
| T1-T0 | 27(3) | 0.15(0.64) | 30(1) | -0.04(0.72) | 0.4504 | 17(3) | 0.17(1.12) | 17(0) | 0.38(1.09) | 0.2029 |
| T2-T0 | 27(3) | 0.37(1.51) | 30(1) | -0.05(0.87) | 0.4811 | 17(3) | 0.14(1.13) | 17(0) | 0.06(0.41) | 0.3674 |
| MMEF |  |  |  |  |  |  |  |  |  |  |
| T0 | 27(3) | 0.94(0.57) | 30(1) | 0.92(0.47) | 0.9681 | 17(3) | 0.28(0.08) | 17(0) | 0.34(0.17) | 0.7040 |
| T1 | 29(1) | 0.78(0.45) | 31(0) | 0.88(0.43) | 0.2167 | 19(1) | 0.33(0.19) | 17(0) | 0.45(0.36) | 0.2533 |
| T2 | 29(1) | 0.91(0.67) | 31(0) | 0.85(0.46) | 0.8301 | 19(1) | 0.32(0.19) | 17(0) | 0.33(0.17) | 0.5570 |
| T1-T0 | 27(3) | -0.21(0.53) | 30(1) | -0.06(0.22) | 0.4793 | 17(3) | 0.06(0.19) | 17(0) | 0.12(0.36) | 0.4942 |
| T2-T0 | 27(3) | -0.06(0.87) | 30(1) | -0.09(0.21) | 0.7854 | 17(3) | 0.04(0.19) | 17(0) | -0.01(0.07) | 0.9723 |

Note. Data are shown as mean±SD. P values were calculated by t-test, signed rank sum test or chi-square test. * p< 0.05. T0: baseline, T1: week 24, T2: week 52.

**Table S14**

Frequency of acute exacerbations between two groups at different time points in subgroup stratified by GOLD grade

| Follow-up | GOLD 1-2 | | | | | GOLD 3-4 | | | | |
| --- | --- | --- | --- | --- | --- | --- | --- | --- | --- | --- |
|  | SHGBZK | | Placebo | | P values | SHGBZK | | Placebo | | P values |
|  | N | Mean (SD) | N | Mean (SD) |  | N | Mean (SD) | N | Mean (SD) |  |
| Baseline | 37(0) | 1.24(0.72) | 39(0) | 1.46(0.76) | 0.1370 | 20(0) | 1.70(0.98) | 16(1) | 2.13(1.36) | 0.2858 |
| Week 24 | 37(0) | 0.02(0.07) | 39(0) | 0.05(0.14) | 0.3038 | 20(0) | 0.02(0.07) | 17(0) | 0.07(0.18) | 0.2351 |
| Week 52 | 37(0) | 0.11(0.39) | 39(0) | 0.41(1.23) | 0.1933 | 20(0) | 0.10(0.45) | 17(0) | 0.71(1.72) | 0.0574 |
| Week 24-52 | 37(0) | 0.01(0.05) | 39(0) | 0.04(0.10) | 0.5415 | 20(0) | 0.01(0.06) | 17(0) | 0.06(0.12) | 0.1177 |

Note. Data are shown as mean±SD. P values were calculated by t-test, signed rank sum test or chi-square test. * p< 0.05. T0: baseline, T1: week 24, T2: week 52.

**Table S15**

Lung function between two groups at different time points in subgroup stratified by gender

| Variable | male | | | | | female | | | | |
| --- | --- | --- | --- | --- | --- | --- | --- | --- | --- | --- |
|  | SHGBZK | | Placebo | | P values | SHGBZK | | Placebo | | P values |
|  | N | Mean (SD) | N | Mean (SD) |  | N | Mean (SD) | N | Mean (SD) |  |
| FEV1 |  |  |  |  |  |  |  |  |  |  |
| T0 | 36(6) | 1.46(0.68) | 37(0) | 1.86(0.70) | 0.0166 | 8(0) | 1.53(0.50) | 10(1) | 1.08(0.37) | 0.0417* |
| T1 | 40(2) | 1.50(0.74) | 37(0) | 1.85(0.63) | 0.0166 | 8(0) | 1.41(0.32) | 11(0) | 1.19(0.53) | 0.3172 |
| T2 | 40(2) | 1.53(0.83) | 37(0) | 1.75(0.65) | 0.0886 | 8(0) | 1.41(0.28) | 11(0) | 1.19(0.54) | 0.3144 |
| T1-T0 | 36(6) | -0.01(0.26) | 37(0) | -0.01(0.26) | 0.7634 | 8(0) | -0.12(0.27) | 10(1) | -0.01(0.12) | 0.3215 |
| T2-T0 | 36(6) | 0.04(0.50) | 37(0) | -0.11(0.20) | 0.1068 | 8(0) | -0.12(0.28) | 10(1) | -0.01(0.09) | 0.3154 |
| FVC |  |  |  |  |  |  |  |  |  |  |
| T0 | 36(6) | 2.95(0.85) | 37(0) | 3.48(0.80) | 0.0080 | 8(0) | 2.47(0.57) | 10(1) | 2.12(0.35) | 0.1292 |
| T1 | 40(2) | 3.00(0.92) | 37(0) | 3.42(0.79) | 0.0370 | 8(0) | 2.45(0.28) | 11(0) | 2.28(0.61) | 0.4288 |
| T2 | 40(2) | 2.92(0.97) | 37(0) | 3.33(0.83) | 0.0520 | 8(0) | 2.37(0.35) | 11(0) | 2.25(0.63) | 0.6466 |
| T1-T0 | 36(6) | -0.04(0.36) | 37(0) | -0.06(0.33) | 0.5440 | 8(0) | -0.02(0.36) | 10(1) | 0.02(0.24) | 0.8239 |
| T2-T0 | 36(6) | -0.09(0.42) | 37(0) | -0.15(0.37) | 0.1868 | 8(0) | -0.10(0.34) | 10(1) | -0.01(0.25) | 1.0000 |
| FEV1% |  |  |  |  |  |  |  |  |  |  |
| T0 | 36(6) | 48.06(13.55) | 37(0) | 52.48(13.47) | 0.1660 | 8(0) | 62.25(12.68) | 10(1) | 49.96(10.66) | 0.0399* |
| T1 | 40(2) | 48.19(13.44) | 37(0) | 53.86(13.12) | 0.0652 | 8(0) | 57.81(11.53) | 11(0) | 51.15(11.45) | 0.2292 |
| T2 | 40(2) | 49.67(13.36) | 37(0) | 51.93(13.43) | 0.4623 | 8(0) | 59.75(9.39) | 11(0) | 51.42(10.29) | 0.3521 |
| T1-T0 | 36(6) | 0.47(6.25) | 37(0) | 1.38(9.59) | 0.8197 | 8(0) | -4.44(11.00) | 10(1) | -0.36(4.68) | 0.0888 |
| T2-T0 | 36(6) | 1.78(10.90) | 37(0) | -0.55(4.52) | 0.6659 | 8(0) | -2.50(9.93) | 10(1) | -0.06(3.63) | 0.6243 |
| PEF |  |  |  |  |  |  |  |  |  |  |
| T0 | 36(6) | 4.23(1.88) | 37(0) | 5.22(2.03) | 0.0208 | 8(0) | 3.56(1.48) | 10(1) | 3.17(1.04) | 0.5199 |
| T1 | 40(2) | 4.47(2.13) | 37(0) | 5.38(1.97) | 0.0331 | 8(0) | 4.04(1.01) | 11(0) | 3.28(1.04) | 0.1294 |
| T2 | 40(2) | 4.68(2.40) | 37(0) | 5.20(2.15) | 0.1767 | 8(0) | 3.83(0.81) | 11(0) | 3.33(1.07) | **0.0364*** |
| T1-T0 | 36(6) | 0.08(0.87) | 37(0) | 0.16(0.97) | 0.3526 | 8(0) | 0.48(0.69) | 10(1) | -0.05(0.39) | 0.2885 |
| T2-T0 | 36(6) | 0.28(1.46) | 37(0) | -0.02(0.82) | 0.9779 | 8(0) | 0.27(0.89) | 10(1) | 0.01(0.31) | 0.4515 |
| MMEF |  |  |  |  |  |  |  |  |  |  |
| T0 | 36(6) | 0.59(0.43) | 37(0) | 0.79(0.50) | 0.0686 | 8(0) | 1.12(0.83) | 10(1) | 0.42(0.22) | 0.0497 |
| T1 | 40(2) | 0.61(0.44) | 37(0) | 0.80(0.45) | 0.0209 | 8(0) | 0.56(0.41) | 11(0) | 0.49(0.37) | 0.4568 |
| T2 | 40(2) | 0.69(0.66) | 37(0) | 0.72(0.47) | 0.2774 | 8(0) | 0.61(0.23) | 11(0) | 0.48(0.37) | 0.1157 |
| T1-T0 | 36(6) | 0.00(0.20) | 37(0) | 0.01(0.33) | 0.6476 | 8(0) | -0.55(0.87) | 10(1) | -0.02(0.05) | 0.3044 |
| T2-T0 | 36(6) | 0.08(0.61) | 37(0) | -0.07(0.20) | 0.2128 | 8(0) | -0.51(0.86) | 10(1) | -0.03(0.06) | 0.3481 |

Note. Data are shown as mean±SD. P values were calculated by t-test, signed rank sum test or chi-square test. * p< 0.05. T0: baseline, T1: week 24, T2: week 52.

**Table S16**

Frequency of acute exacerbations between two groups at different time points in subgroup stratified by gender

| Follow-up | male | | | | | female | | | | |
| --- | --- | --- | --- | --- | --- | --- | --- | --- | --- | --- |
|  | SHGBZK | | Placebo | | P values | SHGBZK | | Placebo | | P values |
|  | N | Mean (SD) | N | Mean (SD) |  | N | Mean (SD) | N | Mean (SD) |  |
| Baseline | 41(1) | 2.02(1.86) | 36(1) | 2.00(1.87) | 0.8225 | 8(0) | 1.88(0.83) | 11(0) | 2.18(1.40) | 0.5895 |
| Week 24 | 42(0) | 0.02(0.07) | 37(0) | 0.05(0.14) | 0.3493 | 8(0) | 0.00(0.00) | 11(0) | 0.03(0.10) | 0.4555 |
| Week 52 | 42(0) | 0.12(0.40) | 37(0) | 0.41(1.24) | 0.2174 | 8(0) | 0.00(0.00) | 11(0) | 0.27(0.65) | 0.2454 |
| Week 24-52 | 42(0) | 0.01(0.04) | 37(0) | 0.03(0.10) | 0.1302 | 8(0) | 0.00(0.00) | 11(0) | 0.04(0.09) | 0.2454 |

Note. Data are shown as mean±SD. P values were calculated by t-test, signed rank sum test or chi-square test. * p< 0.05. T0: baseline, T1: week 24, T2: week 52.
